# “Spatial heterogeneity of environmental risk in randomized prevention trials: consequences and modeling”

**DOI:** 10.1186/s12874-019-0759-z

**Published:** 2019-07-15

**Authors:** Abdoulaye Guindo, Issaka Sagara, Boukary Ouedraogo, Kankoe Sallah, Mahamadoun Hamady Assadou, Sara Healy, Patrick Duffy, Ogobara K. Doumbo, Alassane Dicko, Roch Giorgi, Jean Gaudart

**Affiliations:** 1 Aix Marseille Univ, INSERM, IRD, SESSTIM, Sciences Economiques & Sociales de la Santé & Traitement de l’Information Médicale, Marseille, France; 2 Malaria Research and Training Center – Ogobara K Doumbo, FMOS-FAPH, Mali-NIAID-ICER, Université des Sciences, des Techniques et des Technologies de Bamako, Bamako, Mali; 3 Direction des systèmes d’information en santé, Ministère de la santé, Ouagadougou, Burkina Faso; 4 AP-HP, Hôpital Bichat, Unité de Recherche Clinique PNVS, Paris, France; 5 Laboratory of Malaria Immunology and Vaccinology, National Institute of Allergy and Infectious Diseases, National Institutes of Health, Rockville, Maryland USA; 6 Aix Marseille Univ, APHM, INSERM, IRD, SESSTIM, Hop Timone, BioSTIC, Biostatistic & ICT, Marseille, France

**Keywords:** Randomized prevention trials, Spatial heterogeneity, Stochastic Partial Differential Equation, Integrated Nested Laplace Approximation, Environmental factors

## Abstract

**Background:**

In the context of environmentally influenced communicable diseases, proximity to environmental sources results in spatial heterogeneity of risk, which is sometimes difficult to measure in the field. Most prevention trials use randomization to achieve comparability between groups, thus failing to account for heterogeneity.

This study aimed to determine under what conditions spatial heterogeneity biases the results of randomized prevention trials, and to compare different approaches to modeling this heterogeneity.

**Methods:**

Using the example of a malaria prevention trial, simulations were performed to quantify the impact of spatial heterogeneity and to compare different models.

Simulated scenarios combined variation in baseline risk, a continuous protective factor (age), a non-related factor (sex), and a binary protective factor (preventive treatment). Simulated spatial heterogeneity scenarios combined variation in breeding site density and effect, location, and population density.

The performances of the following five statistical models were assessed: a non-spatial Cox Proportional Hazard (Cox-PH) model and four models accounting for spatial heterogeneity—*i.e.,* a Data-Generating Model, a Generalized Additive Model (GAM), and two Stochastic Partial Differential Equation (SPDE) models, one modeling survival time and the other the number of events. Using a Bayesian approach, we estimated the SPDE models with an Integrated Nested Laplace Approximation algorithm.

For each factor (age, sex, treatment), model performances were assessed by quantifying parameter estimation biases, mean square errors, confidence interval coverage rates (CRs), and significance rates. The four models were applied to data from a malaria transmission blocking vaccine candidate.

**Results:**

The level of baseline risk did not affect our estimates. However, with a high breeding site density and a strong breeding site effect, the Cox-PH and GAM models underestimated the age and treatment effects (but not the sex effect) with a low CR.

When population density was low, the Cox-SPDE model slightly overestimated the effect of related factors (age, treatment). The two SPDE models corrected the impact of spatial heterogeneity, thus providing the best estimates.

**Conclusion:**

Our results show that when spatial heterogeneity is important but not measured, randomization alone cannot achieve comparability between groups. In such cases, prevention trials should model spatial heterogeneity with an adapted method.

**Trial registration:**

The dataset used for the application example was extracted from Vaccine Trial #NCT02334462 (ClinicalTrials.gov registry).

**Electronic supplementary material:**

The online version of this article (10.1186/s12874-019-0759-z) contains supplementary material, which is available to authorized users.

## Background

In the context of communicable diseases such as vector-borne diseases (*e.g.*, malaria, dengue) or other environmentally influenced diseases (*e.g.*, cholera), the location of individuals affects environmental risk. In the case of vector-borne diseases, proximity to an environment favorable to disease transmission (a vector breeding site or an area favorable to mosquito survival) leads to spatial heterogeneity of risk [1]. Most researchers conducting prevention trials consider that randomization is sufficient to achieve comparability between groups, even when they fail to measure environmental risk. Such prevention trials are based on analysis plans that generally fail to account for spatial heterogeneity [2–5]. In some cases, they account for this heterogeneity by using mixed models [6–8], which associate a random spatial effect with a specific spatial scale (country, region, health district, village, etc.), on the assumption that environmental risk is homogeneous on the scale considered and that no spatial interaction occurs between scales (interscale spatial independence). However, spatial heterogeneity of incidence exists on the village scale itself [9], undermining the conditions for applying such models.

Spatial heterogeneity of risk is often difficult to assess. In contexts where disease risk is associated with environmental factors, specific risk factors are not always measurable or can be measured only through in-depth fieldwork. For example, mosquito breeding sites can be small, numerous, and scattered, as is the case for *Anopheles sp.* and *Aedes sp.* (vectors for malaria and dengue, respectively) [10–14]. Similarly, in the context of cholera, it is almost impossible to investigate all water sources (wells or pumps).

While the Cox Proportional Hazard (Cox-PH) model is the most widely used multivariate approach in clinical trials, it is not always applied correctly in contexts of spatial heterogeneity. In view of this, different methods have been proposed that take into account spatial heterogeneity for survival analysis. Generalized Additive Models (GAMs), which were initially developed to model non-linear relationships, are used more and more to model spatial heterogeneity using bivariate spline functions on geographical coordinates as covariates [15–17]. Alternatively, Stochastic Partial Differential Equation (SPDE) models are used to model explicitly outcome variations following the first law of geography [18]. Thus, Lindgren *et al.* have proposed an SPDE model solved using a Gaussian field with a Matèrn covariance function that has good computational properties [19, 20]. Moreover, the SPDE model implemented using the INLA (Integreted Nested Laplace Approximation) algorithm is now used in an ever-wider range of contexts [21–23].

The aim of this study was first to determine under what conditions and to what extent spatial heterogeneity of environmental risk can bias the results of randomized prevention trials, and then to compare the performance of different spatial models in estimating treatment effectiveness. These methods were applied to a malaria vaccine trial conducted in Mali.

## Methods

We simulated 432 scenarios (50 datasets for each scenario, 1,000 individuals for each dataset). These scenarios were analyzed first with a non-spatial Cox-PH model, and then with four models that accounted for spatial heterogeneity, including a Data-Generating model (DGM).

For each factor (age, sex, and treatment), model performance was assessed by quantifying parameter estimation bias, mean square error (MSE), confidence interval coverage rate (CR), and significance rate (SR).

For the main scenarios (32), 500 datasets of 1,000 individuals each were performed for validation (Additional file 1: Figure S1).

### Simulation plan

Event time was simulated using a classic Cox-PH model that accounted for different risks factors (including environmental factors) according to a Weibull distribution. Censoring time was simulated using an exponential distribution.

More precisely, for a given vector of covariates *X*, the instantaneous risk function *λ*_*i*_(*t*, *X*) for individual *i* at time *t* was defined as follows:

$$ {\lambda}_i\left(t,X\right)={\lambda}_i\left(t,{X}^{(i)}\right)+{\lambda}_i\left(t,{X}^{\left(-i\right)}\right) $$

where *λ*_*i*_(*t*, *X*^(*i*)^) was the fixed effect dependent on individual *i* and *λ*_*i*_(*t*, *X*^(−*i*)^) was the spatial effect dependent on the neighborhood of individual *i*.

We then simulated a randomized and controlled prevention trial of *n* = 1,000 individuals in a square area *Ω* of 400 km^2^. Before the treatment randomization was simulated, different risk situations (scenarios) were simulated according to location (spatial distribution of individuals), baseline risk, and heterogeneity of environmental risk (location and density of breeding sites) (see description below). In addition to the treatment effect, two non-spatial risk factors were simulated: one had a significant continuous effect (age factor), and the other had a null effect (sex factor).

The size of datasets and the number of simulations were selected with an accuracy of 0.01 and a variance of 0.09, following standard recommendations [24].

### Location (spatial distribution of individuals)

To simulate the spatial distribution of individuals in the study zone *Ω*, we used an Inhomogeneous Point Process (IPP) in which population density depended on the location of individuals. We considered three clusters, *L*1, *L*2, and *L*3, where the population was at its densest, and we introduced a concentration parameter *τ* to control for population density in these clusters.

The location of these three clusters was randomized using a Homogeneous Point Process (HPP) so as to respect a minimum distance between them (see Additional file 2).

We studied the impact of population density in the three clusters by simulating three different density situations:

*τ* = 0.2 (low population density in the three clusters)

*τ* = 0.5 (average population density in the three clusters)

*τ* = 0.8 (high population density in the three clusters)

### Baseline risk

Baseline risk *λ*_0_ was assumed to be constant for all individuals. It was simulated using a Weibull distribution with a shape parameter set to 1 to obtain a constant value over time. Three baseline risk levels corresponding to three observed epidemiological profiles were used for the scale parameter γ, taking as an example malaria prevalence in Mali [25]:

Low prevalence: *γ* = 6% (*e.g.*, situation in Bamako),

Median prevalence: *γ* = 37% (*e.g.*, situation in Segou),

High prevalence: *γ* = 60% (*e.g.*, situation in Mopti).

### Source of environmental risk (Breeding sites)

Spatial heterogeneity of environmental risk was simulated according to location of breeding site, variation in breeding site effect (defined as Relative Risk *RR*_*b*_), variation in breeding site density, and influence radius of breeding site considered as a constant.

An individual was considered exposed to a breeding site when the distance between this individual and the center of the site was less than the site’s influence radius.

The influence radius of a breeding site was interpreted as the average distance traveled by a mosquito to take a blood meal. The influence radius was set at *r* = 600 m [26], and was constant for all breeding sites.

Considering that breeding site density did not depend on location, we simulated the spatial distribution of breeding sites following a marked HPP where the mark was the breeding site’s influence radius *r.* Thus, an individual could be exposed to zero, one, or more breeding sites, with risk increasing accordingly.

Breeding site density was used to quantify the ratio of the environmental risk area (*i.e.*, the area covered by the influence radius of at least one breeding site) to the total area of the study zone (400 km^2^).

This density was interpreted as the probability of being exposed to at least one breeding site (see Additional file 2). To simulate different scenarios, breeding site density was set at 0.25, 0.5, and 0.75.

Given that breeding site productivity and protection against mosquito bites can be highly variable, the breeding site effect (defined as Relative Risk *RR*_*b*_), was also simulated in four situations:

- Very weak effect (*RR*_*b*_ = 1.05),
- Weak effect (*RR*_*b*_ = 1.20),
- Strong effect (*RR*_*b*_ = 1.5),
- Very strong effect (*RR*_*b*_ = 3).

### Treatment

Allocation to control and intervention groups was simulated according to a symmetric Bernoulli distribution $\mathcal{B}\left(n,0.5\right)$ using individual randomization and without accounting for location or for other factors.

Subsequently, four levels of treatment effect (defined as Relative Risk *RR*_*t*_) were simulated:

- Very weak effect (*RR*_*t*_ = 0.95),
- Weak effect (*RR*_*t*_ = 0.8),
- Strong effect (*RR*_*t*_ = 0.6),
- Very strong effect (*RR*_*t*_ = 0.25).

Indeed, while the effectiveness of prevention trials (including malaria-prevention trials) is highly variable, in most cases it falls within the relative risk range of 0.25 to 0.95 [27–32].

### Other risk factors

Although the aim of our study was to assess the impact of spatial heterogeneity of environmental risk, we included two additional factors in our analysis: one was related to the disease (age), and the other was not (sex). These two factors were independent of spatial location, and their effect was constant over time.

Thus, a binary variable identified as the sex factor was simulated using a symmetrical binomial distribution with a null effect (defined as Relative Risk *RR*_*s*_ = 1).

$$ Sex\sim \mathcal{B}\ \left(n,p=0.5\ \right) $$

A continuous disease-related variable identified as the age factor was simulated following a piecewise uniform distribution, based on the population distribution by age group in Mali [33]. The protective effect of this variable (defined as Relative Risk *RR*_*a*_), which was taken from the literature [25], was fixed and constant over time (*RR*_*a*_ = 0.84):

$$ \mathrm{Age}1\sim \mathcal{U}\ \left(47.3\%\mathrm{n},\left[0.25,14\right]\right) $$

$$ \mathrm{Age}2\sim \mathcal{U}\ \left(19.2\%\mathrm{n},\right]14,24\left]\right) $$

$$ \mathrm{Age}3\sim \mathcal{U}\ \left(26.8\%\mathrm{n},\Big]24,54\Big]\right) $$

$$ \mathrm{Age}4\sim \mathcal{U}\ \left(3.76\%\mathrm{n},\Big]54,64\Big]\right) $$

$$ \mathrm{Age}5\sim \mathcal{U}\ \left(2.94\%\mathrm{n},\right]64,75\left]\right) $$

### Events and Censoring

Event time *T*_*event*_ was simulated using a classic Cox-PH model that accounted for the different risk factors detailed above, namely baseline risk, age, sex, treatment, and environmental risk (breeding sites). The instantaneous risk function of individual *i* was calculated as follows:

*λ*_*i*_(*t*| *X*) = *λ*_0_ *exp* (*β*_1_*Age*_*i*_ + *β*_2_*Sex*_*i*_ + *β*_3_*Treatment*_*i*_ + *β*_4_*BS*_*i*_)  [equation 1]

*λ*_0_ was the baseline risk considered constant over time, *BS*_*i*_ was the number of breeding sites to which individual *i* was exposed, and *β*_1_, *β*_2_, *β*_3_, *β*_4_ were the parameters associated with the covariates age, sex, treatment, and breeding site, respectively.

Censoring time *T*_*cens*_ was simulated following an exponential distribution. Thus, observation time *T* was defined as the minimum between censoring time and event time.

$$ T=\mathit{\min}\left({T}_{event},{T}_{cens}\right) $$

**Fig. 1:**
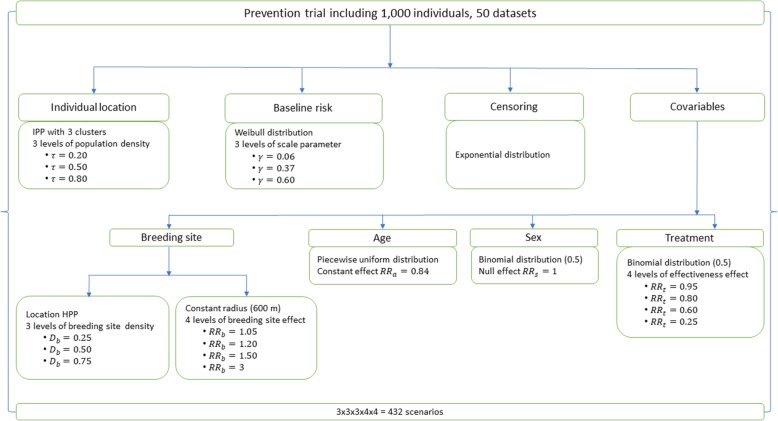
Simulation scheme for the different scenarios

The combination of these different simulation parameters resulted in 432 scenarios, each containing 50 datasets (Figure 1). Datasets of 1,000 individuals each yielded a power greater than 85% when the treatment effect (*RR*_*t*_) was less than 0.8. For a treatment effect set at *RR*_*t*_ = 0.95, power was around 65%.

**Fig. 2:**
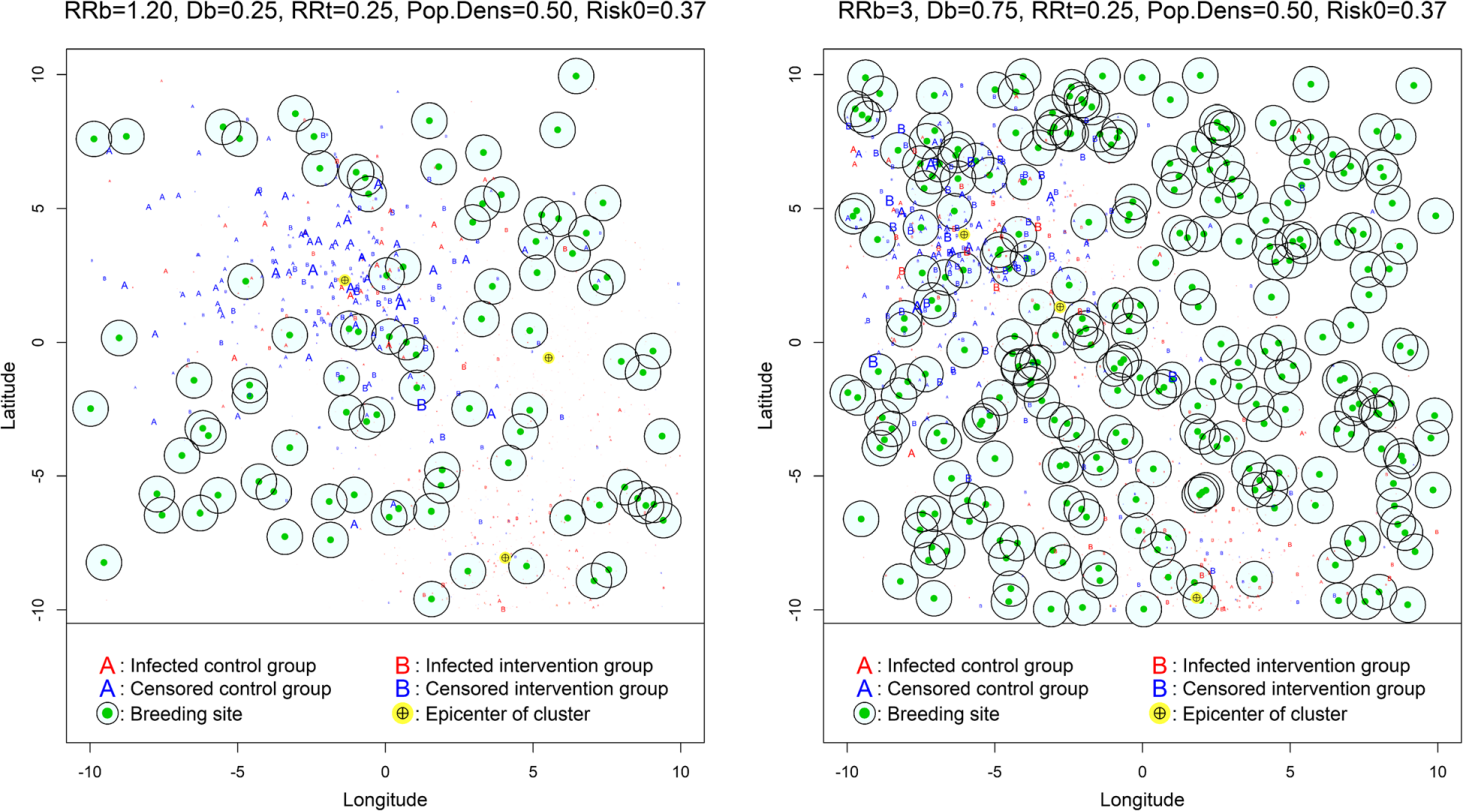
Structure of simulated data (the size of the points is proportional to survival time)

An example of the simulated data structure is shown in Figure 2.

### Model Evaluation and Comparison

For each scenario, we analyzed each dataset using five statistical models (see Additional file 1 for details):

a DGM, according to equation 1;

Model Evaluation and Comparisona classic non-spatial Cox-PH model, whose parameters were estimated with the maximum likelihood method, according to an equation similar to equation 1 but without taking into account the environmental risk factor [34, 35];

a GAM model, also with proportional hazards, which modeled the spatial effect with a bivariate spline function [36] (as detailed below);

two SPDE models (as detailed below), which modeled spatial heterogeneity using a Gaussian field with a Matèrn covariance function: one modeled survival time with a Weibull distribution (Cox-SPDE), and the other modeled the number of events with a Poisson distribution (P-SPDE).

Using a Bayesian approach, we estimated the SPDE models (Cox-SPDE and P-SPDE) with the INLA algorithm to optimize computation times [37, 38].

### Generalized Additive Model (GAM)

The GAM model was originally designed to study non-linear links [39], usually with spline functions. In our particular case, a bivariate spline function of the geographic coordinates of individuals (latitude and longitude) was used to account for spatial heterogeneity of environmental risk [36, 40]:

$$ \lambda \left(t,X\right)={\lambda}_0(t)\exp \left({\beta}_1{X}_1+{\beta}_2{X}_2+\dots +{\beta}_n{X}_n+f\left( long, lat\right)\right) $$

for each point data, *f* was a bivariate spline function modeling the spatial effect, *long* was longitude, and *lat* was latitude.

### SPDE model

The SPDE model was originally designed to model spatial structures using a Gaussian field [41]. This Bayesian model is generally written as follows:

$$ Y\mid \beta, X,Z\sim \mathbb{P}\left(Y|\mu, \phi \right) $$

$$ Z\sim GF\left(0,\varSigma \right) $$

ℙ is the distribution of *Y* (dependent variable), *X* is the vector of covariates (fixed effects), *Z* is a Gaussian field (*GF*) with a Matèrn covariance function *Σ* (random spatial effect), and *μ* = *E*(*Y*) and *ϕ* are the parameters of the distribution of *Y*, *μ* = *E*(*Y*) = *h*(*βX* + *Z*), where *h* is the canonical link function.

The Matèrn covariance function is used to control for spatial dependence and for the regularity of the Gaussian field (see Additional file 2). Other covariance functions, including exponential ones, are particular cases of the Matèrn covariance function [42]. In our particular case, we applied the SPDE model in two different ways to model outcome distribution: We applied a spatial Cox-PH model (Cox-SPDE) to model survival time, and a Poisson model (P-SPDE) to discretize survival time and to model the number of events within each time interval (Poisson distribution).

### Cox-SPDE model

The Cox-SPDE model is the classic Cox-PH model to which a Gaussian field is added to model the spatial structure [38, 41]. In our particular case, the dependent variable (*Y*) was survival time:

$$ \lambda \left(t,X\right)={\lambda}_0(t)\exp \left({\beta}_1{X}_1+{\beta}_2{X}_2+\dots +{\beta}_n{X}_n+Z\right) $$

### P-SPDE model

The P-SPDE model is a piecewise exponential model driven by a counting process [41, 43, 44]. Thus, for a number of events *D* (where *δ*_*ik*_ is the indicator of the event in individual *i* = 1, 2, … , *n* in the time interval *k* = 1, 2, … , *D*, and where *T*_*ik*_ is survival time in individual *i* in the *k*^*th*^ interval), *δ* is assumed to follow a Poisson distribution of parameter *π* (average number of events per time interval).

In our particular case, the spatial structure was modeled with a Gaussian field, as earlier described. The dependent variable was the number of events per time interval instead of survival time.

$$ \delta \sim Poisson\ \left(\pi \right) $$

$$ \log \left(\pi \right)={\lambda}_0+{\beta}_1{X}_1+{\beta}_2{X}_2+\dots +{\beta}_n{X}_n+\log (T)+Z $$

### Model performance measures

The parameter *β* represented the true effect of each covariate estimated by $\hat{\beta}$, with a standard deviation $\overline{Sd\left(\hat{\beta}\right)}$estimated on *K* datasets for each scenario and with a 95% Confidence Interval *CI*(*β*).

The models were compared using four performance measures [24, 45]: estimation bias $B\left(\hat{\beta}\right)$, mean square error (MSE), coverage rate (CR), and significance rate (SR), as defined in Additional file 2.

### Application

The four models (Cox-PH, GAM, Cox-SPDE, P-SPDE) were applied, according to the above specifications, to data from a vaccine trial aimed at testing a malaria transmission blocking vaccine candidate: Pfs230 [46]. This randomized controlled trial was conducted in 2015-2016 in the town of Bancoumana, Mali (for more details, see [47]). In our study, the event of interest was a clinical malaria episode. The age, sex, and geographical coordinates (GPS) of participants were also collected. The preventive fraction of the vaccine was calculated based on an estimated prevalence of 77%.

## Results

### Data-Generating Model (DGM)

The DGM was the simulation model, which took into account environmental risk. As expected, biases and MSEs were almost null and nearly always had the desired CR, regardless of the scenario (Additional file 3).

### Impact of baseline risk on estimates

The baseline risk level had roughly no impact on the performance measures of the different models, independently of other parameters (Additional file 4: Figure S2). For example, with a weak breeding site effect (Relative Risk *RR*_*b*_ = 1.05), a low breeding site density (*D*_*b*_ = 0.25), a moderate treatment effect (Relative Risk *RR*_*t*_ = 0.6), and a low population density (*τ* = 0.2), performance measures were identical for a baseline risk of 0.06 and a baseline risk of 0.6 for both the Cox-PH model (bias = -9.37 10^-3^ and -8.14 10^-3^, respectively) and the Cox-SPDE model (bias = -26.57 10^-3^ and -21.38 10^-3^, respectively).

Similarly, for the age and sex effects, performance measures were little impacted by changes in baseline risk, regardless of the scenario.

The following results were obtained with a baseline risk of 0.37.

### Estimates of the treatment effect

When the breeding site effect was weak (Relative Risk *RR*_*b*_ ≤ 1.2), breeding site density had little impact on the bias and MSE of the treatment effect, which were low for all models, regardless of the scenario.

**Fig. 3:**
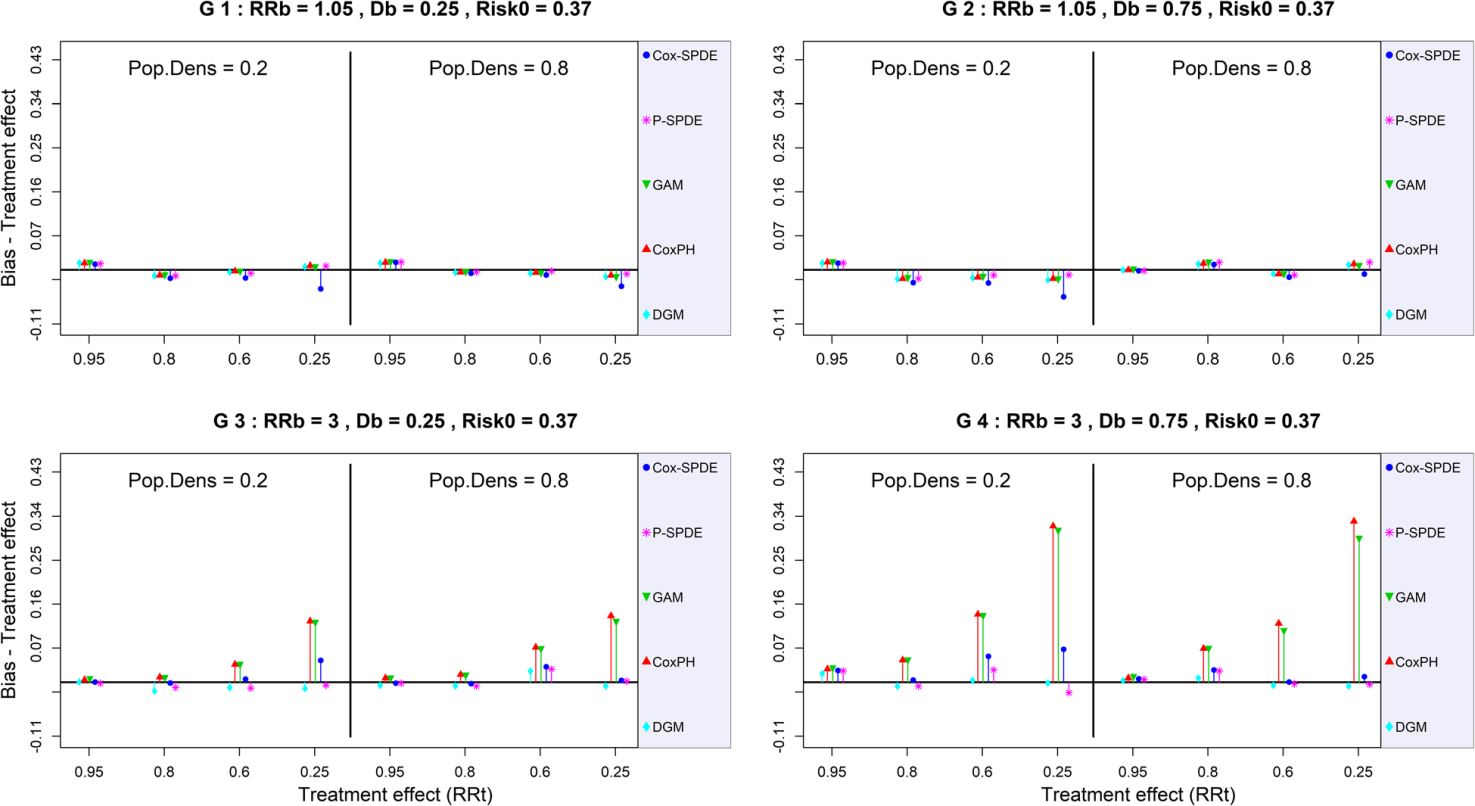
Bias of the treatment effect with a baseline risk of 0.37 DGM: Data-Generating Model, Cox-PH: Cox Proportional Hazard model, GAM: Generalized Additive Model, Cox-SPDE: Cox Stochastic Partial Differential Equation Model, P-SPDE: Poisson Stochastic Partial Differential Equation, RRb: Breeding site Relative Risk, Db: Breeding site Density, RRt: Treatment Relative Risk, Pop.Dens: Population Density, Risk0: Baseline Risk

Thus, with a weak breeding site effect (*RR*_*b*_ ≤ 1.2), the bias of the treatment effect ranged from -0.034 to 0.045 for all models except the Cox-SPDE model, regardless of the scenario (G1 and G2 in Figures 3 and Additional file 5: Figure S3).

Under these conditions of weak breeding site effect, the Cox-SPDE model tended to slightly overestimate the treatment effect when population density was low (*τ* = 0.2), all the more so when the treatment effect was strong (Table 1). For example, with *RR*_*t*_ = 0.25, *RR*_*b*_ = 1.05, *D*_*b*_ = 0.25, and *τ* = 0.2, the Cox-SPDE model overestimated the treatment effect with a maximum bias of -0.071.By contrast, with a high population density (*τ* = 0.8, other parameters remaining unchanged), the bias of the treatment effect was -0.033. Yet even under conditions of low population density, this overestimation was corrected when the treatment effect was weak (*RR*_*t*_ ≥ 0.80). For example, for the Cox-SPDE model, with *RR*_*b*_ = 1.05, *D*_*b*_ = 0.25, and *τ* = 0.2, the bias of the treatment effect varied between -0.011 and 0.012 for *RR*_*t*_ = 0.95.

**Table 1:** Model performance measures for estimation of the treatment effect (baseline risk 0.37)

RRb	RRt	Model	Breeding site density											
			0.25						0.75					
			Population density						Population density					
			0.2			0.8			0.2			0.8		
			Bias	CR	SR	Bias	CR	SR	Bias	CR	SR	Bias	CR	SR
1.05	0.95	Cox-PH	0.014	0.96	0.06	0.015	0.88	0.08	0.016	0.92	0.06	|< 0.001|	0.96	0.04
		GAM	0.014	0.98	0.06	0.015	0.86	0.08	0.015	0.92	0.06	|< 0.001|	0.94	0.04
		Cox-SPDE	0.012	0.98	0.08	0.016	0.86	0.08	0.014	0.94	0.06	-0.002	0.96	0.04
		P-SPDE	0.013	0.98	0.08	0.016	0.86	0.1	0.014	0.94	0.06	-0.002	0.96	0.04
	0.80	Cox-PH	-0.011	0.98	0.74	-0.005	0.98	0.92	-0.018	0.96	0.92	0.013	0.96	0.76
		GAM	-0.012	0.98	0.74	-0.006	0.98	0.9	-0.018	0.96	0.92	0.014	0.96	0.74
		Cox-SPDE	-0.017	0.94	0.74	-0.007	0.98	0.9	-0.026	0.94	0.92	0.011	0.96	0.76
		P-SPDE	-0.012	0.96	0.76	-0.004	0.98	0.9	-0.018	0.96	0.9	0.016	0.96	0.76
	0.60	Cox-PH	-0.003	0.84	1.00	-0.005	0.94	1.00	-0.015	0.94	1.00	-0.008	0.94	1.00
		GAM	-0.004	0.88	1.00	-0.008	0.94	1.00	-0.015	0.96	1.00	-0.01	0.94	1.00
		Cox-SPDE	-0.017	0.8	1.00	-0.011	0.94	1.00	-0.027	0.96	1.00	-0.015	0.94	1.00
		P-SPDE	-0.007	0.88	1.00	-0.002	0.94	1.00	-0.011	0.98	1.00	-0.011	0.96	1.00
	0.25	Cox-PH	0.009	0.98	1.00	-0.011	0.98	1.00	-0.018	0.96	1.00	0.012	0.9	1.00
		GAM	0.005	0.96	1.00	-0.016	0.96	1.00	-0.02	0.96	1.00	0.007	0.92	1.00
		Cox-SPDE	-0.039	0.9	1.00	-0.033	0.92	1.00	-0.055	0.9	1.00	-0.008	0.9	1.00
		P-SPDE	0.008	0.94	1.00	-0.008	0.96	1.00	-0.01	0.96	1.00	0.016	0.96	1.00
3	0.95	Cox-PH	0.005	1.00	0.02	0.009	0.94	0.06	0.027	0.96	0.04	0.009	0.94	0.06
		GAM	0.005	1.00	0.02	0.007	0.94	0.08	0.027	0.96	0.04	0.01	0.98	0.08
		Cox-SPDE	|< 0.001|	1.00	0.02	-0.002	0.94	0.08	0.024	0.92	0.04	0.007	1.00	0.04
		P-SPDE	-0.002	1.00	0.02	-0.002	0.94	0.08	0.023	0.9	0.04	0.006	1.00	0.04
	0.80	Cox-PH	0.01	0.96	0.78	0.016	0.92	0.8	0.045	0.92	0.6	0.069	0.84	0.5
		GAM	0.008	0.98	0.78	0.013	0.92	0.8	0.044	0.94	0.58	0.068	0.86	0.5
		Cox-SPDE	-0.002	0.98	0.78	-0.003	0.96	0.82	0.004	1.00	0.64	0.025	0.98	0.48
		P-SPDE	-0.011	0.98	0.8	-0.008	0.96	0.82	-0.008	1.00	0.64	0.023	1.00	0.5
	0.60	Cox-PH	0.036	0.9	1.00	0.072	0.86	1.00	0.139	0.62	1.00	0.121	0.64	1.00
		GAM	0.035	0.92	1.00	0.067	0.86	1.00	0.135	0.6	1.00	0.104	0.78	1.00
		Cox-SPDE	0.006	0.96	1.00	0.032	0.94	1.00	0.053	0.92	1.00	|< 0.001|	0.96	1.00
		P-SPDE	-0.012	0.96	1.00	0.027	0.92	1.00	0.025	0.92	1.00	-0.003	0.96	1.00
	0.25	Cox-PH	0.125	0.78	1.00	0.136	0.58	1.00	0.32	0.02	1.00	0.33	0.06	1.00
		GAM	0.121	0.78	1.00	0.123	0.68	1.00	0.309	0.02	1.00	0.293	0.1	1.00
		Cox-SPDE	0.045	0.94	1.00	0.004	0.98	1.00	0.067	0.94	1.00	0.011	0.94	1.00
		P-SPDE	-0.006	1.00	1.00	0.002	1.00	1.00	-0.021	0.98	1.00	-0.004	0.92	1.00

*CR* Coverage Rate, *SR* Significance Rate, *Cox-PH* Cox Proportional Hazard model, *GAM* Generalized Additive Model, *Cox-SPDE* Cox-Stochastic Partial Differential Equation Model, *P-SPDE* Poisson-Stochastic Partial Differential Equation Model, *RRt* Treatment Relative Risk, *RRb* Breeding site Relative Risk. See Additional file 6 for *MSE* Mean Square Error

**Fig. 4:**
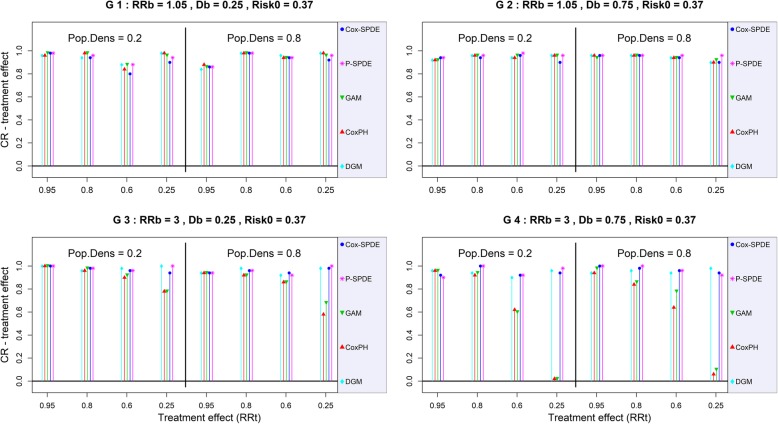
CR of the treatment effect with a baseline risk of 0.37. DGM: Data-Generating Model, Cox-PH: Cox Proportional Hazard model, GAM: Generalized Additive Model, Cox-SPDE: Cox Stochastic Partial Differential Equation Model, P-SPDE: Poisson Stochastic Partial Differential Equation, RRb: Breeding site Relative Risk, Db: Breeding site Density, RRt: Treatment Relative Risk, Pop.Dens: Population Density, Risk0: Baseline Risk.

**Fig. 5:**
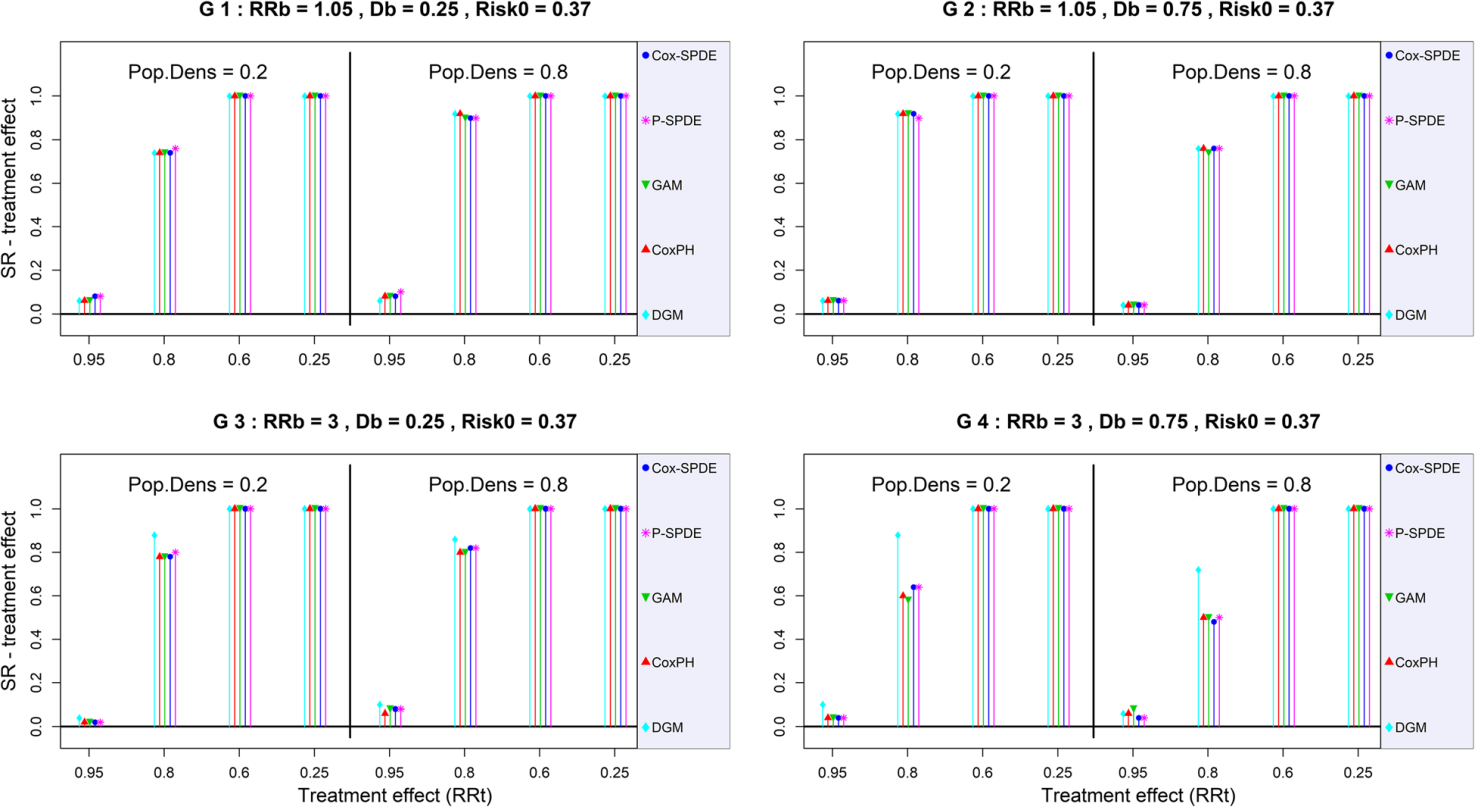
SR of the treatment effect with a baseline risk of 0.37. DGM: Data-Generating Model, Cox-PH: Cox Proportional Hazard model, GAM: Generalized Additive Model, Cox-SPDE: Cox Stochastic Partial Differential Equation Model, P-SPDE: Poisson Stochastic Partial Differential Equation, RRb: Breeding site Relative Risk, Db: Breeding site Density, RRt: Treatment Relative Risk, Pop.Dens: Population Density, Risk0: Baseline Risk.

The SR (*resp.* CR) of the treatment effect was similar between all models regardless of the weak breeding site effect scenario. It ranged from 2% to 24% (*resp.* from 86% to 100%) for *RR*_*t*_ = 0.95, and reached 100% (*resp.* from 82% to 100%) for *RR*_*t*_ = 0.25. As expected, there was a lack of power when the treatment effect was weak (*RR*_*t*_ = 0.95) (G1 and G2 in Figures 4 and 5).

By contrast, when the breeding site effect was strong (*RR*_*b*_ ≥ 1.51), the increase in breeding site density caused an important increase in the bias of the treatment effect for the Cox-PH and GAM models. The bias of the treatment effect was even higher when this effect was strong (G3 and G4 in Figure 3).

For example, for the Cox-PH model, with *RR*_*b*_ = 3, *D*_*b*_ = 0.25, and *τ* = 0.2, the bias of the treatment effect could reach a maximum of 0.139 for the highest level of treatment effect (*RR*_*t*_ = 0.25).

With these same parameters, the bias of the treatment effect reached a maximum of 0.131 for the GAM model. As for the two SPDE models, the bias of the treatment effect reached a maximum of 0.054 for the Cox-SPDE model and a maximum of 0.010 for the P-SPDE model.

With a higher breeding site density (*D*_*b*_ = 0.75, other parameters remaining unchanged), the bias of the treatment effect reached a maximum of 0.342 for the Cox-PH model for the highest level of treatment effect. The bias of the treatment effect reached a maximum of 0.33 for the GAM model. As for the two SPDE models, the bias of the treatment effect reached a maximum of 0.09 for the Cox-SPDE model and a maximum of 0.014 for the P-SPDE model.

When the breeding site effect was strong (*RR*_*b*_ ≥ 1.51), the CR of the treatment effect was negatively impacted by an increase in breeding site density for the Cox-PH and GAM models (G3 and G4 in Figures 4 and 5). This impact was even greater when the treatment effect was strong.

For example, with a strong breeding site effect *RR*_*b*_ = 3 and a high breeding site density (*D*_*b*_ = 0.75), the maximum CR of the treatment effect varied between 94% to 8% for the Cox-PH model, between 92% to 14% for the GAM model, between 98% to 96% for the Cox-SPDE model, and between 100% to 96% for the P-SPDE model.

The SR of the treatment effect was not impacted by strong breeding site effects. It remained similar between all models, but varied from one scenario to the next depending on the level of treatment effect (with an expected lack of power for the weak treatment effect *RR*_*t*_ = 0.95). Indeed, for the highest level of treatment effect (*RR*_*t*_ = 0.25), the SR of the treatment effect varied between 98% and 100% for all models.

### Application

In the context of the Pfs230 vaccine trial, none of the models yielded a significant vaccine effect on clinical episodes. The Cox-SPDE model estimated a slightly higher preventive fraction (29.26% [-10.93%; 51.21%]), followed closely by the P-SPDE model (26.95% [-15.25%; 49.90%]). By contrast, the non-spatial Cox-PH model estimated a slightly lower preventive fraction (24.64% [-16.56%; 47.66%]), as did the GAM model (22.33% [-22.25%; 46.82%]).

### Estimates of the age effect

When the breeding site effect was weak (*RR*_*b*_ = 1.05 or 1.2), breeding site density had little impact on the bias and MSE of the age effect, regardless of the model. For example, regardless of the weak breeding site effect scenario, the bias of the age effect was around -0.0035 for all models except the Cox-SPDE model (G1 and G2 in Additional file 7: Figure S4 and Additional file 8: Figure S5). As was observed for the treatment effect, the latter model tended to slightly overestimate the age effect, all the more so when population density in the clusters was low. Thus, for a population density *τ* = 0.2, the bias was around -0.007 (Table 2).

**Table 2:** Model performance measures for estimation of the age effect (baseline risk 0.37)

RRb	RRt	Model	Breeding site density											
			0.25						0.75					
			Population density						Population density					
			0.2			0.8			0.2			0.8		
			Bias	CR	SR	Bias	CR	SR	Bias	CR	SR	Bias	CR	SR
1.05	0.95	Cox-PH	0.002	0.96	1.00	|< 0.001|	0.9	1.00	-0.002	0.98	1.00	-0.001	0.98	1.00
		GAM	0.001	0.94	1.00	-0.001	0.9	1.00	-0.002	0.96	1.00	-0.002	0.96	1.00
		Cox-SPDE	-0.004	0.76	1.00	-0.003	0.86	1.00	-0.006	0.72	1.00	-0.003	0.88	1.00
		P-SPDE	|< 0.001|	0.94	1.00	-0.001	0.96	1.00	-0.001	0.96	1.00	-0.001	0.92	1.00
	0.80	Cox-PH	|< 0.001|	0.96	1.00	-0.001	0.96	1.00	|< 0.001|	0.96	1.00	-0.002	0.84	1.00
		GAM	|< 0.001|	0.96	1.00	-0.002	0.96	1.00	-0.001	0.98	1.00	-0.003	0.88	1.00
		Cox-SPDE	-0.004	0.76	1.00	-0.003	0.86	1.00	-0.006	0.68	1.00	-0.005	0.72	1.00
		P-SPDE	-0.001	0.96	1.00	-0.001	0.92	1.00	|< 0.001|	0.94	1.00	-0.002	0.92	1.00
	0.60	Cox-PH	0.001	0.9	1.00	-0.001	0.96	1.00	-0.002	0.96	1.00	0.001	0.96	1.00
		GAM	|< 0.001|	0.92	1.00	-0.002	0.96	1.00	-0.002	0.94	1.00	|< 0.001|	0.96	1.00
		Cox-SPDE	-0.003	0.84	1.00	-0.003	0.86	1.00	-0.007	0.66	1.00	-0.002	0.88	1.00
		P-SPDE	|< 0.001|	0.94	1.00	-0.001	0.94	1.00	-0.002	0.98	1.00	|< 0.001|	0.94	1.00
	0.25	Cox-PH	|< 0.001|	0.98	1.00	-0.001	0.98	1.00	-0.001	0.94	1.00	|< 0.001|	0.96	1.00
		GAM	|< 0.001|	0.98	1.00	-0.003	0.9	1.00	-0.002	0.92	1.00	|< 0.001|	0.94	1.00
		Cox-SPDE	-0.006	0.66	1.00	-0.004	0.8	1.00	-0.006	0.76	1.00	-0.003	0.8	1.00
		P-SPDE	|< 0.001|	0.96	1.00	-0.001	0.96	1.00	|< 0.001|	0.94	1.00	|< 0.001|	1.00	1.00
3	0.95	Cox-PH	0.018	0.16	1.00	0.016	0.32	1.00	0.041	0.00	1.00	0.042	0.00	1.00
		GAM	0.017	0.2	1.00	0.014	0.5	1.00	0.04	0.00	1.00	0.037	0.00	1.00
		Cox-SPDE	0.007	0.74	1.00	|< 0.001|	0.98	1.00	0.01	0.84	1.00	0.003	0.86	1.00
		P-SPDE	-0.001	0.92	1.00	-0.001	0.96	1.00	|< 0.001|	0.94	1.00	|< 0.001|	0.96	1.00
	0.80	Cox-PH	0.018	0.18	1.00	0.019	0.18	1.00	0.042	0.00	1.00	0.041	0.00	1.00
		GAM	0.017	0.26	1.00	0.018	0.32	1.00	0.04	0.00	1.00	0.036	0.00	1.00
		Cox-SPDE	0.007	0.86	1.00	0.005	0.9	1.00	0.011	0.8	1.00	0.002	0.98	1.00
		P-SPDE	0.001	0.92	1.00	0.001	0.96	1.00	0.002	0.9	1.00	|< 0.001|	0.96	1.00
	0.60	Cox-PH	0.017	0.26	1.00	0.016	0.3	1.00	0.042	0.00	1.00	0.04	0.00	1.00
		GAM	0.016	0.28	1.00	0.015	0.52	1.00	0.041	0.00	1.00	0.037	0.00	1.00
		Cox-SPDE	0.007	0.8	1.00	0.001	0.94	1.00	0.012	0.76	1.00	0.001	0.96	1.00
		P-SPDE	0.001	0.98	1.00	-0.001	0.98	1.00	0.002	0.94	1.00	-0.001	0.96	1.00
	0.25	Cox-PH	0.018	0.2	1.00	0.016	0.28	1.00	0.042	0.00	1.00	0.043	0.00	1.00
		GAM	0.017	0.2	1.00	0.014	0.44	1.00	0.041	0.00	1.00	0.038	0.00	1.00
		Cox-SPDE	0.007	0.8	1.00	-0.001	0.94	1.00	0.011	0.78	1.00	0.003	0.92	1.00
		P-SPDE	0.001	0.96	1.00	-0.001	0.92	1.00	|< 0.001|	0.98	1.00	0.001	0.94	1.00

*CR* Coverage Rate, *SR* Significance Rate, *Cox-PH* Cox Proportional Hazard model, *GAM* Generalized Additive Model, *Cox-SPDE* Cox-Stochastic Partial Differential Equation Model, *P-SPDE* Poisson-Stochastic Partial Differential Equation Model, *RRt* Treatment Relative Risk, *RRb* Breeding site Relative Risk. See Additional file 6 for *MSE* Mean Square Error

The SR of the age effect was identical for all models regardless of the scenario. However, the CR of the age effect was slightly lower for the Cox-SPDE model when population density was low. Indeed, with a weak breeding site effect (*RR*_*b*_ = 1.05), a low breeding site density (*D*_*b*_ = 0.25), and a population density *τ* = 0.2, the CR of the age effect ranged from 86% to 100% for the non-spatial Cox-PH model and from 62% to 92% for the Cox-SPDE model.

Conversely, when the breeding site effect was strong (*RR*_*b*_ = 1.51 and 3), the bias of the age effect increased markedly with breeding site density *D*_*b*_ for the Cox-PH and GAM models (G3 and G4 in Additional file 7: Figure S4 and Additional file 8: Figure S5). For example, for the Cox-PH model, with a strong breeding site effect (*RR*_*b*_ = 3) and a low population density (*τ* = 0.2), the bias of the age effect was around 0.017 for a low breeding site density (*D*_*b*_ = 0.25) and around 0.041 for a high breeding site density (*D*_*b*_ = 0.75).

The bias of the age effect was very similar between the GAM model and the Cox-PH model, regardless of the scenario. For the Cox-SPDE model, the bias of the age effect was slightly impacted by population density: it was lower when this density was high. With a strong breeding site effect (*RR*_*b*_ = 3) and a high breeding site density (*D*_*b*_ = 0.75), the bias of the age effect was around 0.01 for a low population density (*τ* = 0.2) and around 0.001 for a high population density (*τ* = 0.8). For the P-SPDE model, the bias of the age effect was low regardless of the scenario (~0). It should be noted that the bias of the age effect was similar between the P-SPDE model and the Cox-SPDE model, all the more so when population density was high.

The CR of the age effect was low for the Cox-PH and GAM models (G3 and G4 in Additional file 9: Figure S6). For example, for the Cox-PH model (*resp.* GAM model), with a strong breeding site effect (*RR*_*b*_ = 3), the CR of the age effect was 0% (*resp.* < 2%) when breeding site density was high (*D*_*b*_ = 0.75). Under these same conditions, for the Cox-SPDE and P-SPDE models, the CR of the age effect remained high at 82% to 100%, respectively. However, the SR of the age effect was 100% for all models, regardless of the scenario (Additional file 10: Figure S7).

It is important to note that for these simulated datasets, the level of treatment effect had no impact on the estimates of the age effect, regardless of the scenario.

### Estimates of the sex effect

Regardless of the scenario, the bias, MSE, CR, and SR of the sex effect were roughly identical for all models (Table 3), with very little variation from one scenario to the next (Additional file 11: Figure S8, Additional file 12: Figure S9, Additional file 13: Figure S10, Additional file 14: Figure S11).

**Table 3:** Model performance measures for estimation of the sex effect (baseline risk 0.37)

RRb	RRt	Model	Breeding site density											
			0.25						0.75					
			Population density						Population density					
			0.2			0.8			0.2			0.8		
			Bias	CR	SR	Bias	CR	SR	Bias	CR	SR	Bias	CR	SR
1.05	0.95	Cox-PH	-0.009	1.00	0.00	-0.022	0.88	0.12	-0.013	0.96	0.04	0.004	0.92	0.08
		GAM	-0.009	1.00	0.00	-0.021	0.88	0.12	-0.013	0.96	0.04	0.005	0.92	0.08
		Cox-SPDE	-0.009	1.00	0.00	-0.024	0.88	0.12	-0.015	0.96	0.04	0.006	0.96	0.04
		P-SPDE	-0.008	1.00	0.00	-0.024	0.88	0.12	-0.014	0.96	0.04	0.005	0.96	0.04
	0.80	Cox-PH	-0.01	0.96	0.04	|< 0.001|	0.96	0.04	0.012	0.96	0.04	0.011	0.92	0.08
		GAM	-0.01	0.98	0.02	|< 0.001|	0.98	0.02	0.013	0.96	0.04	0.012	0.92	0.08
		Cox-SPDE	-0.01	0.96	0.04	0.001	0.98	0.02	0.014	0.96	0.04	0.013	0.94	0.06
		P-SPDE	-0.011	0.96	0.04	|< 0.001|	0.98	0.02	0.013	0.96	0.04	0.012	0.94	0.06
	0.60	Cox-PH	-0.024	0.98	0.02	0.004	0.98	0.02	0.002	0.92	0.08	|< 0.001|	0.94	0.06
		GAM	-0.024	0.98	0.02	0.003	0.98	0.02	0.003	0.92	0.08	0.002	0.94	0.06
		Cox-SPDE	-0.023	0.96	0.04	0.004	0.98	0.02	0.001	0.96	0.04	0.003	0.94	0.06
		P-SPDE	-0.023	0.96	0.04	0.005	0.98	0.02	0.001	0.98	0.02	0.003	0.94	0.06
	0.25	Cox-PH	-0.004	0.96	0.04	0.007	0.98	0.02	-0.006	0.94	0.06	-0.001	0.96	0.04
		GAM	-0.003	0.96	0.04	0.006	0.98	0.02	-0.007	0.94	0.06	-0.001	0.96	0.04
		Cox-SPDE	-0.005	0.96	0.04	0.008	0.98	0.02	-0.008	0.94	0.06	|< 0.001|	0.94	0.06
		P-SPDE	-0.005	0.96	0.04	0.008	0.98	0.02	-0.007	0.94	0.06	|< 0.001|	0.96	0.04
3	0.95	Cox-PH	0.002	0.98	0.02	-0.019	0.92	0.08	-0.026	0.92	0.08	0.003	0.96	0.04
		GAM	0.002	0.98	0.02	-0.019	0.9	0.1	-0.028	0.92	0.08	0.004	0.96	0.04
		Cox-SPDE	0.002	0.96	0.04	-0.022	0.9	0.1	-0.039	0.96	0.04	0.013	0.94	0.06
		P-SPDE	0.003	0.96	0.04	-0.022	0.9	0.1	-0.042	0.94	0.06	0.014	0.94	0.06
	0.80	Cox-PH	0.004	0.94	0.06	-0.008	0.94	0.06	-0.006	0.96	0.04	-0.004	0.96	0.04
		GAM	0.005	0.94	0.06	-0.008	0.94	0.06	-0.009	0.94	0.06	-0.001	0.94	0.06
		Cox-SPDE	0.005	0.92	0.08	-0.009	0.98	0.02	-0.01	0.96	0.04	0.002	0.96	0.04
		P-SPDE	0.006	0.92	0.08	-0.009	0.96	0.04	-0.01	0.96	0.04	0.003	0.96	0.04
	0.60	Cox-PH	-0.016	0.98	0.02	0.002	0.98	0.02	-0.01	0.96	0.04	-0.007	0.9	0.1
		GAM	-0.018	0.98	0.02	0.003	0.96	0.04	-0.01	0.98	0.02	-0.004	0.92	0.08
		Cox-SPDE	-0.018	0.98	0.02	0.001	0.94	0.06	-0.014	0.96	0.04	-0.001	0.96	0.04
		P-SPDE	-0.018	0.98	0.02	0.001	0.92	0.08	-0.013	0.92	0.08	-0.001	0.96	0.04
	0.25	Cox-PH	-0.02	0.9	0.1	-0.003	0.98	0.02	-0.001	0.92	0.08	-0.006	0.92	0.08
		GAM	-0.018	0.92	0.08	-0.001	0.98	0.02	|< 0.001|	0.9	0.1	0.002	0.92	0.08
		Cox-SPDE	-0.015	0.9	0.1	-0.003	0.96	0.04	0.007	0.96	0.04	-0.002	0.92	0.08
		P-SPDE	-0.015	0.9	0.1	-0.004	0.98	0.02	0.009	0.96	0.04	-0.002	0.9	0.1

*CR* Coverage Rate, *SR* Significance Rate, *Cox-PH* Cox Proportional Hazard model, *GAM* Generalized Additive Model, *Cox-SPDE* Cox-Stochastic Partial Differential Equation Model, *P-SPDE* Poisson-Stochastic Partial Differential Equation Model, *RRt* Treatment Relative Risk, *RRb* Breeding site Relative Risk. See Additional file 6 for *MSE* Mean Square Error

## Discussion

The aim of this study was to highlight the impact of the spatial heterogeneity of non-measured environmental risk on the results of prevention trials by using simulated data in different scenarios. We have shown that despite randomization, spatial heterogeneity leads to underestimating the treatment effect, with a CR that is almost null in some situations. In our study, the conditions leading to the most biased results were strong environmental effect (breeding site effect in our application context) in conjunction with high environmental density (breeding site density in our application context).

This is unsurprising, as environmental risk is known to vary according to the location of individuals and to environmental factors [48]. Bias correction was achieved with models that accounted for the location of individuals as a proxy for variation in environmental risk, in particular with the P-SPDE model.

The limits of the breeding site effect *RR*_*b*_were set between 1.05 and 3 and those of breeding site density *D*_*b*_ (which expressed the probability of being exposed to at least one breeding site) were set between 0.25 and 0.75. Bias increased linearly, and was important starting from *RR*_*b*_ = 1.5, even when breeding site density was low (*D*_*b*_ = 0.25). However, scenarios with a high breeding site density (*D*_*b*_ = 0.75) and a weak breeding site effect (*RR*_*b*_ = 1.05, 1.20) did not yield a clear bias.

Despite randomization, the treatment effect also impacted the estimates. Indeed, bias increased linearly with the treatment effect, except when the breeding site effect was weak (*RR*_*b*_ ≤ 1.2). With a strong treatment effect (Relative Risk *RR*_*t*_ ≤ 0.6), a strong breeding site effect (*RR*_*b*_ ≥ 1.5), and a high breeding site density (*D*_*b*_ = 0.75), underestimation was maximal and CR was almost null.

Neither baseline risk nor population density had a strong impact on the quality of the estimates. The range of values selected for the baseline risk was within the limits observed in our application context (malaria). However, the absence of an effect of baseline risk was expected because baseline risk applied uniformly to the entire study zone, without influencing the estimates.

In our application context, environmental risk depended little on population density; it depended mainly on breeding site density, as breeding sites are the source of malaria vectors. Our results would likely have been different if the studied pathology had been highly dependent on population density (*e.g.*, cholera).

As expected, no significant effect was observed in the context of the vaccine trial. The absence of a significant vaccine effect on malaria episodes may explain why the difference in estimates between the different models was small. However, these estimates were consistent with our simulations results, as the effects estimated with the Cox-PH and GAM models were weaker than those estimated with the Cox-SPDE and P-SPDE models.

In the literature, spatial heterogeneity is usually accounted for with classic mixed models. While these models were not explicitly developed to account for spatial heterogeneity, they can partly capture spatial heterogeneity by aggregating individuals with the same risk profile. One such model is the Structured Additive Regression (STAR) model [49–52], which takes into account both the contiguity structure and the spatial correlation between areas. In this model, the study zone is split into several predefined subdivisions to represent spatial heterogeneity of environmental risk, and environmental risk is then assumed to be homogeneous within each subdivision [7, 53]. However, as our study indicates, two close individuals can have different risks depending on the proximity of the risk source, which means that the assumption of homogeneity is not always tenable. This is especially the case when administrative subdivisions are used, or when the studied disease is strongly associated with the environment—for instance malaria, for which fine-scale heterogeneity of environmental risk has been described [9]. When the study zone is split even more finely, the excessive number of subdivisions to be included in the mixed model leads to an inflation in the number of parameters to be estimated, and the estimate of variation within each overly small subdivision becomes unstable. In other words, this approach involves a choice between the homogeneity hypothesis and the number of parameters, and the yielded results depend on the choice of shape and size of the subdivisions. In the context of vector-borne diseases, the construction of homogeneous risk areas would require observing every single breeding site, which is unrealistic [10]. The aim of our study, then, was not to account for a particular measured spatial structure (to which a STAR model could be applied), but to highlight the impact of spatial structure on study results and to propose different approaches for modeling spatial heterogeneity when spatial risk factors are not precisely measured. Although few studies have accounted for spatial heterogeneity of environmental risk, many have examined temporal variation in risk.

One method used to study this variation is the Cox-PH model, in which the duration of the study is stratified into time intervals so as to obtain periods of homogeneous risk [54, 55]. However, more continuous approaches (especially ones using spline functions) have also been used [56]. An increasing number of studies have used GAM models to account for spatial heterogeneity of risk [39, 57]. In this approach, spatial heterogeneity is modeled with a bivariate spline function of the geographic coordinates of individuals [36]. However, in our study, little difference in performance was found between the GAM model and the Cox-PH model. This may be explained by the fact that the GAM model estimates local risk by aggregating individual data [40, 56], when in fact survival approaches (such as the one we used) require that these data be kept separate. Moreover, even when the true spatial sources of environmental risk (breeding sites) are not measured, we know that their location differs from that of individuals. This inaccuracy, along with the mode of estimation of bivariate spline functions, may explain the poor results yielded in our study by the GAM model [38, 41]. Our best results were obtained with the Cox-SPDE and P-SPDE models, which modeled the spatial effect using a Gaussian field (random spatial effect) with a Matèrn covariance function. In our simulation study, the P-SPDE model remained stable and followed closely the DGM model, regardless of the scenario. However, the Cox-SPDE model was a little sensitive to population density, slightly overestimating parameters when population density was low. Lastly, as the Matèrn function decreased with distance, the spatial effect on survival time became very small. The difference in results between the two SPDE models for low population densities may therefore be explained by the fact that the P-SPDE model was used to model the number of events as opposed to survival time. It should be noted, however, that both models showed better results than the classic Cox-PH model.

## Conclusion

Our study shows that bias due to spatial heterogeneity of environmental risk is not adequately eliminated with randomization. The underestimation of the treatment effect (with almost null CRs in some situations) highlighted in our study may explain why certain treatments or strategies against malaria end up being rejected. Spatial location modeling can reduce bias due to spatial heterogeneity of environmental risk, the latter being sometimes difficult to measure. For this purpose, SPDE models that model spatial heterogeneity with a Gaussian field seem to be the most appropriate.

## Additional files

Additional file 1: **Figure S1.** Bias of the treatment effect with a baseline risk of 0.37 for 500 simulations. DGM: Data-Generating Model, Cox-PH: Cox Proportional Hazard model, GAM: Generalized Additive Model, Cox-SPDE: Cox-Stochastic Partial Differential Equation Model, P-SPDE: Poisson-Stochastic Partial Differential Equation, RRb: Breeding site Relative Risk, Db: Breeding site Density, RRt: Treatment Relative Risk, Pop.Dens: Population Density, Risk0: Baseline Risk. (TIF 782 kb)

Additional file 2: Description of mathematical formulas and terms used in this manuscript. (DOCX 17 kb)

Additional file 3: Data-Generating Model (DGM) performance index for estimating the different factor effects (baseline risk 0.37). (DOCX 23 kb)

Additional file 4: **Figure S2.** Bias of the treatment effect with a population density of 0.5. DGM: Data-Generating Model, Cox-PH: Cox Proportional Hazard model, GAM: Generalized Additive Model, Cox-SPDE: Cox-Stochastic Partial Differential Equation Model, P-SPDE: Poisson-Stochastic Partial Differential Equation, RRb: Breeding site Relative Risk, Db: Breeding site Density, RRt: Treatment Relative Risk, Pop.Dens: Population Density, Risk0: Baseline Risk. (TIF 773 kb)

Additional file 5: **Figure S3.** MSE of the treatment effect with a baseline risk of 0.37. DGM: Data-Generating Model, Cox-PH: Cox Proportional Hazard model, GAM: Generalized Additive Model, Cox-SPDE: Cox-Stochastic Partial Differential Equation Model, P-SPDE: Poisson-Stochastic Partial Differential Equation, RRb: Breeding site Relative Risk, Db: Breeding site Density, RRt: Treatment Relative Risk, Pop.Dens: Population Density, Risk0: Baseline Risk. (TIF 777 kb)

Additional file 6: MSE of the treatment, age, and sex effect for all models (baseline risk 0.37). (DOCX 32 kb)

Additional file 7: **Figure S4.** Bias of the age effect with a baseline risk of 0.37. DGM: Data-Generating Model, Cox-PH: Cox Proportional Hazard model, GAM: Generalized Additive Model, Cox-SPDE: Cox-Stochastic Partial Differential Equation Model, P-SPDE: Poisson-Stochastic Partial Differential Equation, RRb: Breeding site Relative Risk, Db: Breeding site Density, RRt: Treatment Relative Risk, Pop.Dens: Population Density, Risk0: Baseline Risk. (TIF 794 kb)

Additional file 8: **Figure S5.** MSE of the age effect with a baseline risk of 0.37.DGM: Data-Generating Model, Cox-PH: Cox Proportional Hazard model, GAM: Generalized Additive Model, Cox-SPDE: Cox-Stochastic Partial Differential Equation Model, P-SPDE: Poisson-Stochastic Partial Differential Equation, RRb: Breeding site Relative Risk, Db: Breeding site Density, RRt: Treatment Relative Risk, Pop.Dens: Population Density, Risk0: Baseline Risk. (TIF 753 kb)

Additional file 9: **Figure S6.** CR of the age effect with a baseline risk of 0.37. DGM: Data-Generating Model, Cox-PH: Cox Proportional Hazard model, GAM: Generalized Additive Model, Cox-SPDE: Cox-Stochastic Partial Differential Equation Model, P-SPDE: Poisson-Stochastic Partial Differential Equation, RRb: Breeding site Relative Risk, Db: Breeding site Density, RRt: Treatment Relative Risk, Pop.Dens: Population Density, Risk0: Baseline Risk. (TIF 1247 kb)

Additional file 10: **Figure S7**. SR of the age effect with a baseline risk of 0.37. DGM: Data-Generating Model, Cox-PH: Cox Proportional Hazard model, GAM: Generalized Additive Model, Cox-SPDE: Cox-Stochastic Partial Differential Equation Model, P-SPDE: Poisson-Stochastic Partial Differential Equation, RRb: Breeding site Relative Risk, Db: Breeding site Density, RRt: Treatment Relative Risk, Pop.Dens: Population Density, Risk0: Baseline Risk. (TIF 1407 kb)

Additional file 11: **Figure S8.** Bias of the sex effect with a baseline risk of 0.37. DGM: Data-Generating Model, Cox-PH: Cox Proportional Hazard model, GAM: Generalized Additive Model, Cox-SPDE: Cox-Stochastic Partial Differential Equation Model, P-SPDE: Poisson-Stochastic Partial Differential Equation, RRb: Breeding site Relative Risk, Db: Breeding site Density, RRt: Treatment Relative Risk, Pop.Dens: Population Density, Risk0: Baseline Risk. (TIF 820 kb)

Additional file 12: **Figure S9.** MSE of the sex effect with a baseline risk of 0.37. DGM: Data-Generating Model, Cox-PH: Cox Proportional Hazard model, GAM: Generalized Additive Model, Cox-SPDE: Cox-Stochastic Partial Differential Equation Model, P-SPDE: Poisson-Stochastic Partial Differential Equation, RRb: Breeding site Relative Risk, Db: Breeding site Density, RRt: Treatment Relative Risk, Pop.Dens: Population Density, Risk0: Baseline Risk. (TIF 1147 kb)

Additional file 13: **Figure S10**. CR of the sex effect with a baseline risk of 0.37. DGM: Data-Generating Model, Cox-PH: Cox Proportional Hazard model, GAM: Generalized Additive Model, Cox-SPDE: Cox-Stochastic Partial Differential Equation Model, P-SPDE: Poisson-Stochastic Partial Differential Equation, RRb: Breeding site Relative Risk, Db: Breeding site Density, RRt: Treatment Relative Risk, Pop.Dens: Population Density, Risk0: Baseline Risk. (TIF 1380 kb)

Additional file 14: **Figure S11.** MSE of the treatment effect with the classic and bootstrap methods. DGM: Data-Generating Model, Cox-PH: Cox Proportional Hazard model, GAM: Generalized Additive Model, Cox-SPDE: Cox-Stochastic Partial Differential Equation Model, P-SPDE: Poisson-Stochastic Partial Differential Equation, RRb: Breeding site Relative Risk, Db: Breeding site Density, RRt: Treatment Relative Risk, Pop.Dens: Population Density, Risk0: Baseline Risk. (TIF 650 kb)

## Data Availability

The dataset used and/or analyzed in the present study is available from the corresponding author on reasonable request.

## Abbreviations

Cox-PH: Cox Proportional Hazard model
Cox-SPDE: Cox-Stochastic Partial Differential Equation
CR: Coverage Rate
GAM: Generalized Additive Model
HPP: Homogeneous Point Process
INLA: Integrated Nested Laplace Approximation
IPP: Inhomogeneous Point Process
MSE: Mean Square Error
Pop.Dens: Population Density
P-SPDE: Poisson-Stochastic Partial Differential Equation
Risk0: Baseline Risk
SPDE: Stochastic Partial Differential Equation
SR: Significance Rate
