# Supplementary figures and images for: “Spatial heterogeneity of environmental risk in randomized prevention trials: consequences and modeling”
Source: BMC Med Res Methodol. 2019 Jul 15;19:149. doi: 10.1186/s12874-019-0759-z (PMC6632226; doi:10.1186/s12874-019-0759-z)

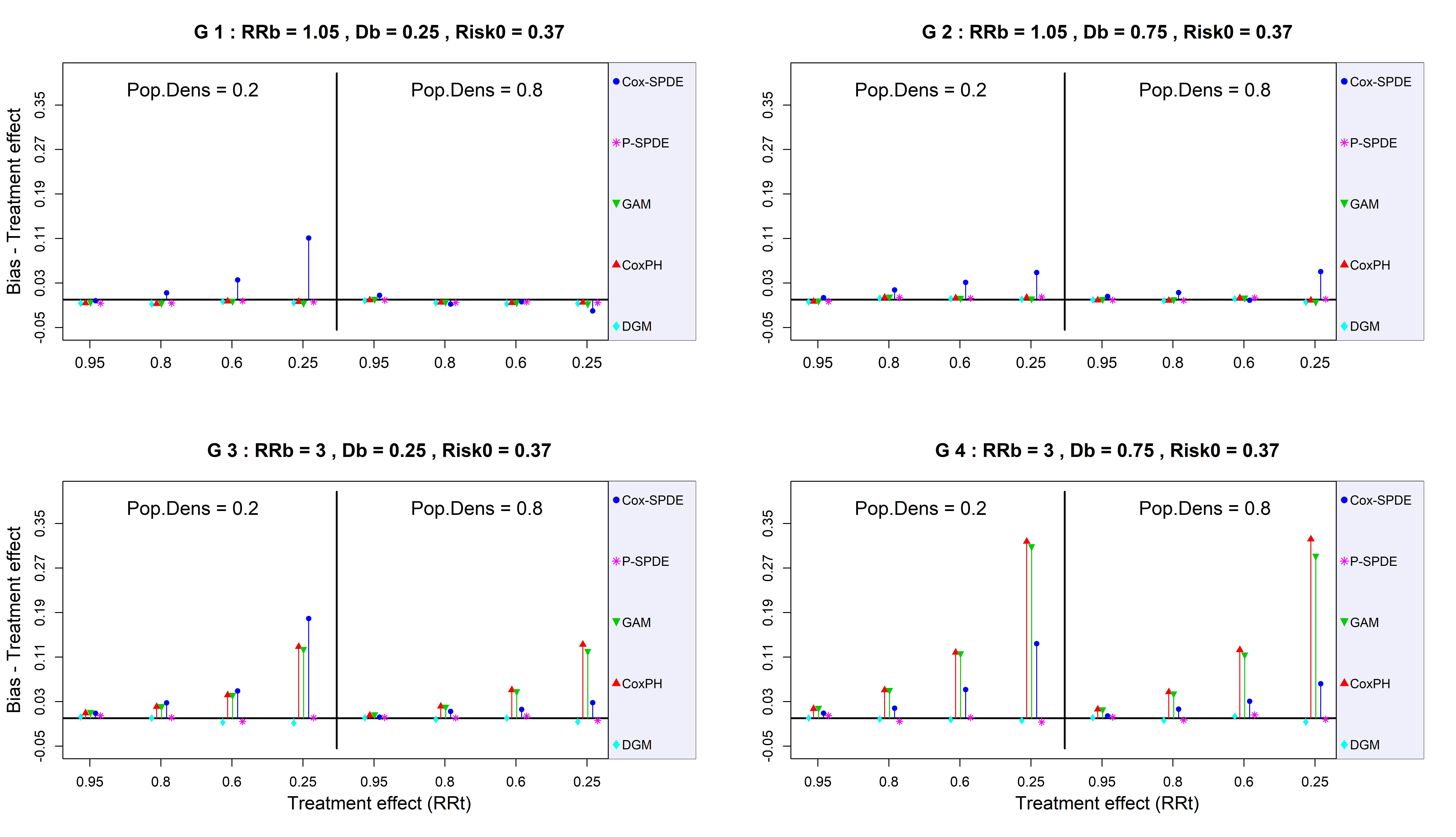

Supplement: Supplementary file 1 — Figure S1. Bias of the treatment effect with a baseline risk of 0.37 for 500 simulations. DGM: Data-Generating Model, Cox-PH: Cox Proportional Hazard model, GAM: Generalized Additive Model, Cox-SPDE: Cox-Stochastic Partial Differential Equation Model, P-SPDE: Poisson-Stochastic Partial Differential Equation, RRb: Breeding site Relative Risk, Db: Breeding site Density, RRt: Treatment Relative Risk, Pop.Dens: Population Density, Risk0: Baseline Risk. (TIF 782 kb) [file 12874_2019_759_MOESM1_ESM.tif]

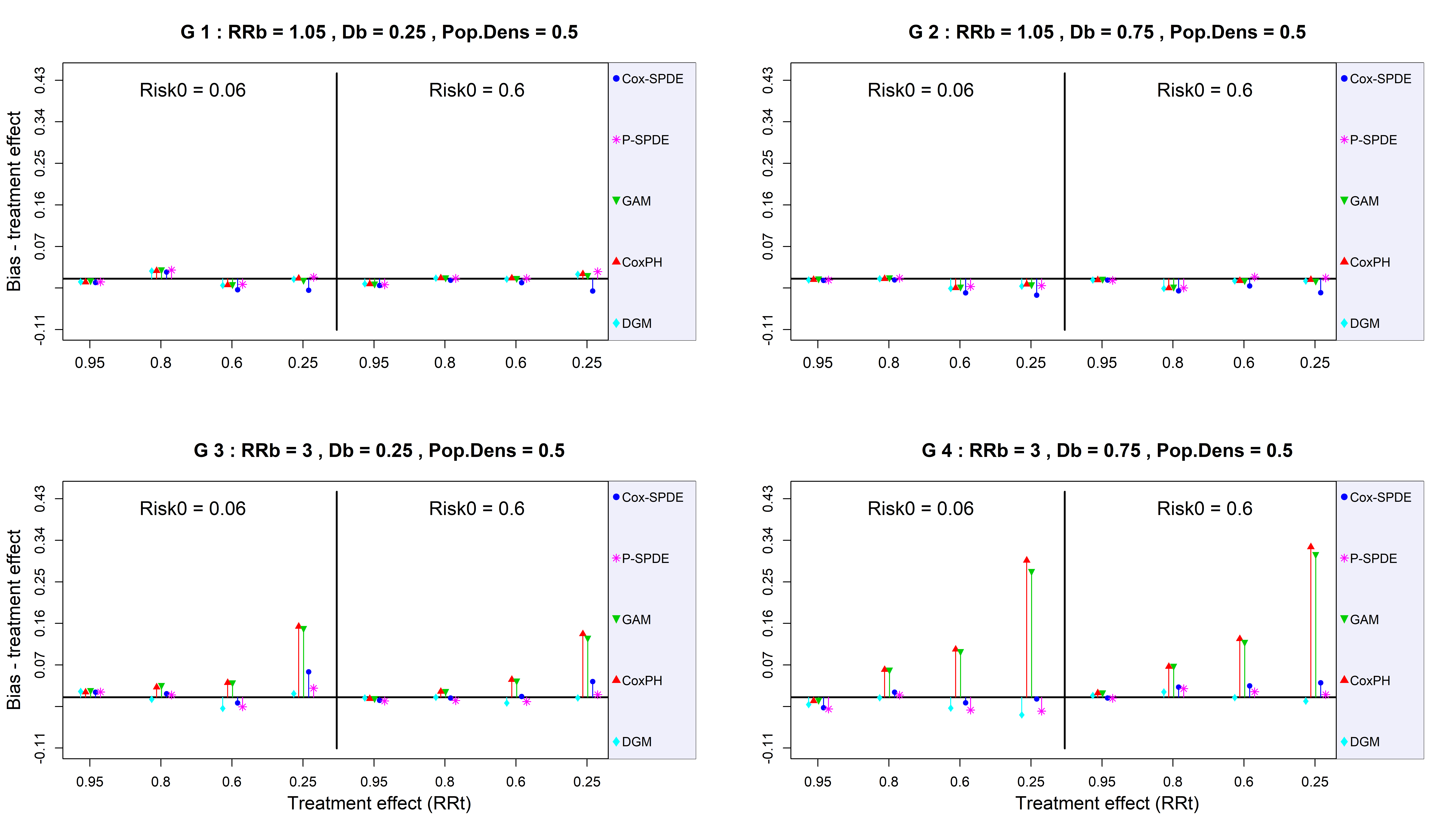

Supplement: Supplementary file 4 — Figure S2. Bias of the treatment effect with a population density of 0.5. DGM: Data-Generating Model, Cox-PH: Cox Proportional Hazard model, GAM: Generalized Additive Model, Cox-SPDE: Cox-Stochastic Partial Differential Equation Model, P-SPDE: Poisson-Stochastic Partial Differential Equation, RRb: Breeding site Relative Risk, Db: Breeding site Density, RRt: Treatment Relative Risk, Pop.Dens: Population Density, Risk0: Baseline Risk. (TIF 773 kb) [file 12874_2019_759_MOESM4_ESM.tif]

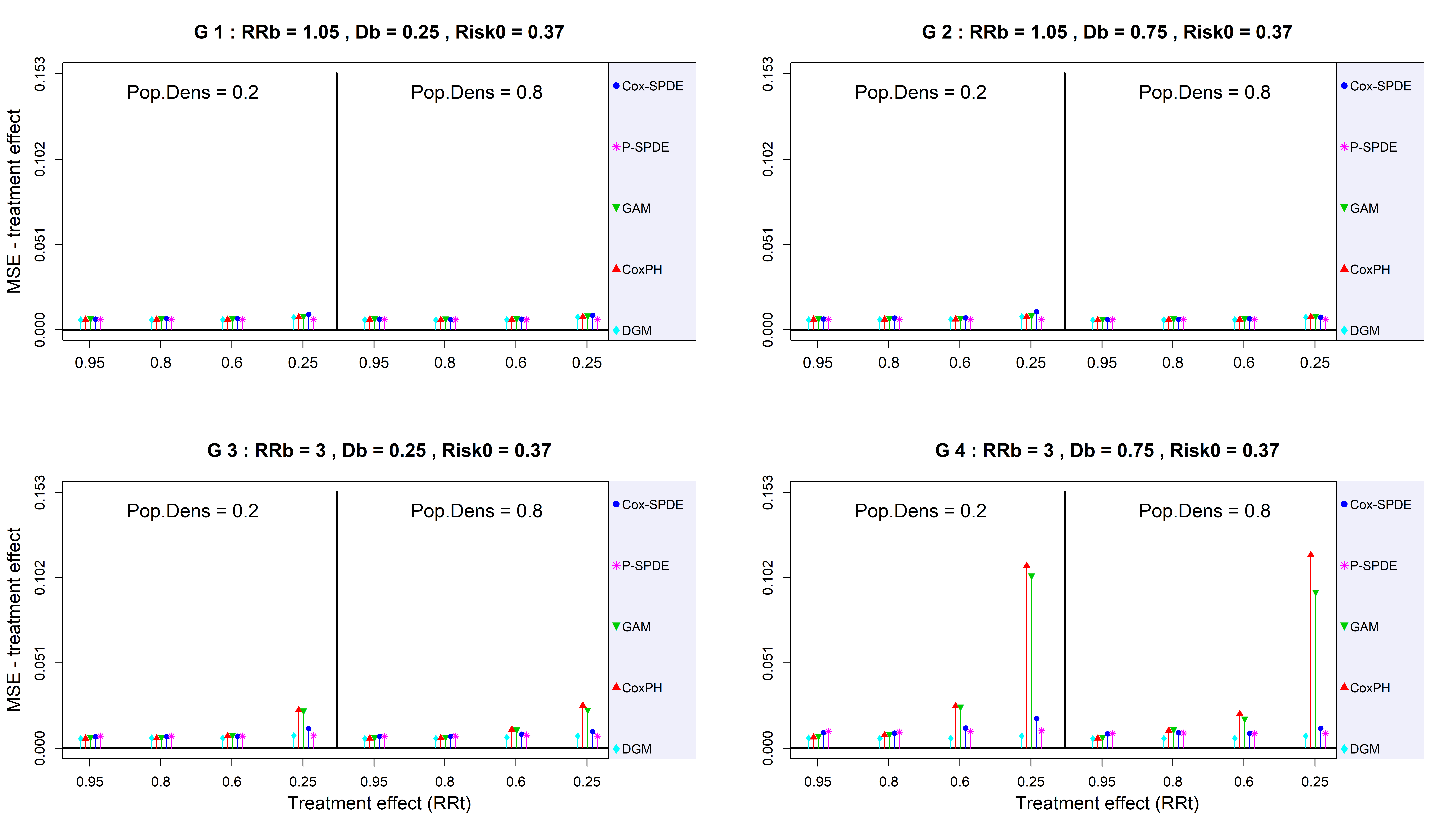

Supplement: Supplementary file 5 — Figure S3. MSE of the treatment effect with a baseline risk of 0.37. DGM: Data-Generating Model, Cox-PH: Cox Proportional Hazard model, GAM: Generalized Additive Model, Cox-SPDE: Cox-Stochastic Partial Differential Equation Model, P-SPDE: Poisson-Stochastic Partial Differential Equation, RRb: Breeding site Relative Risk, Db: Breeding site Density, RRt: Treatment Relative Risk, Pop.Dens: Population Density, Risk0: Baseline Risk. (TIF 777 kb) [file 12874_2019_759_MOESM5_ESM.tif]

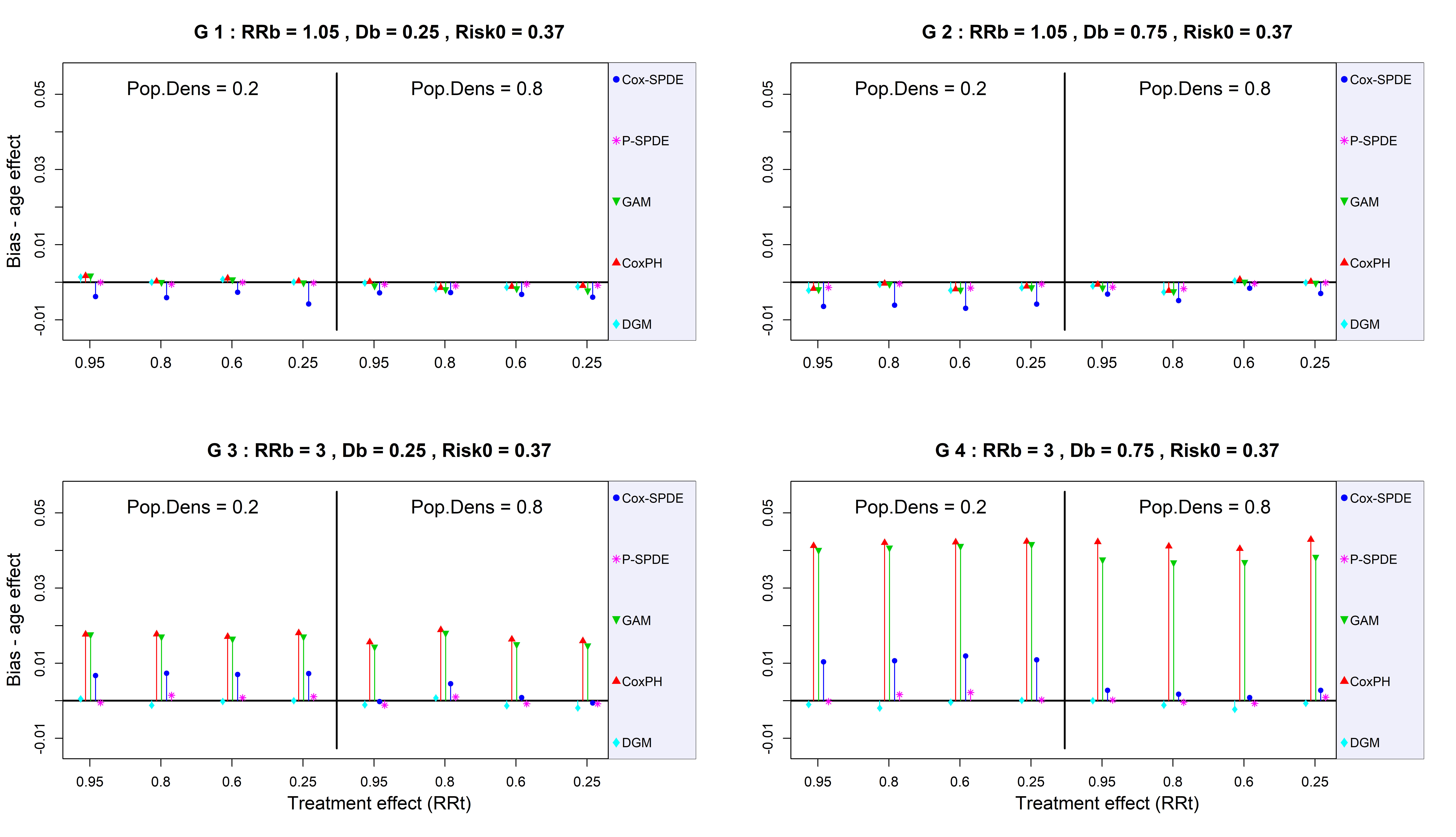

Supplement: Supplementary file 7 — Figure S4. Bias of the age effect with a baseline risk of 0.37. DGM: Data-Generating Model, Cox-PH: Cox Proportional Hazard model, GAM: Generalized Additive Model, Cox-SPDE: Cox-Stochastic Partial Differential Equation Model, P-SPDE: Poisson-Stochastic Partial Differential Equation, RRb: Breeding site Relative Risk, Db: Breeding site Density, RRt: Treatment Relative Risk, Pop.Dens: Population Density, Risk0: Baseline Risk. (TIF 794 kb) [file 12874_2019_759_MOESM7_ESM.tif]

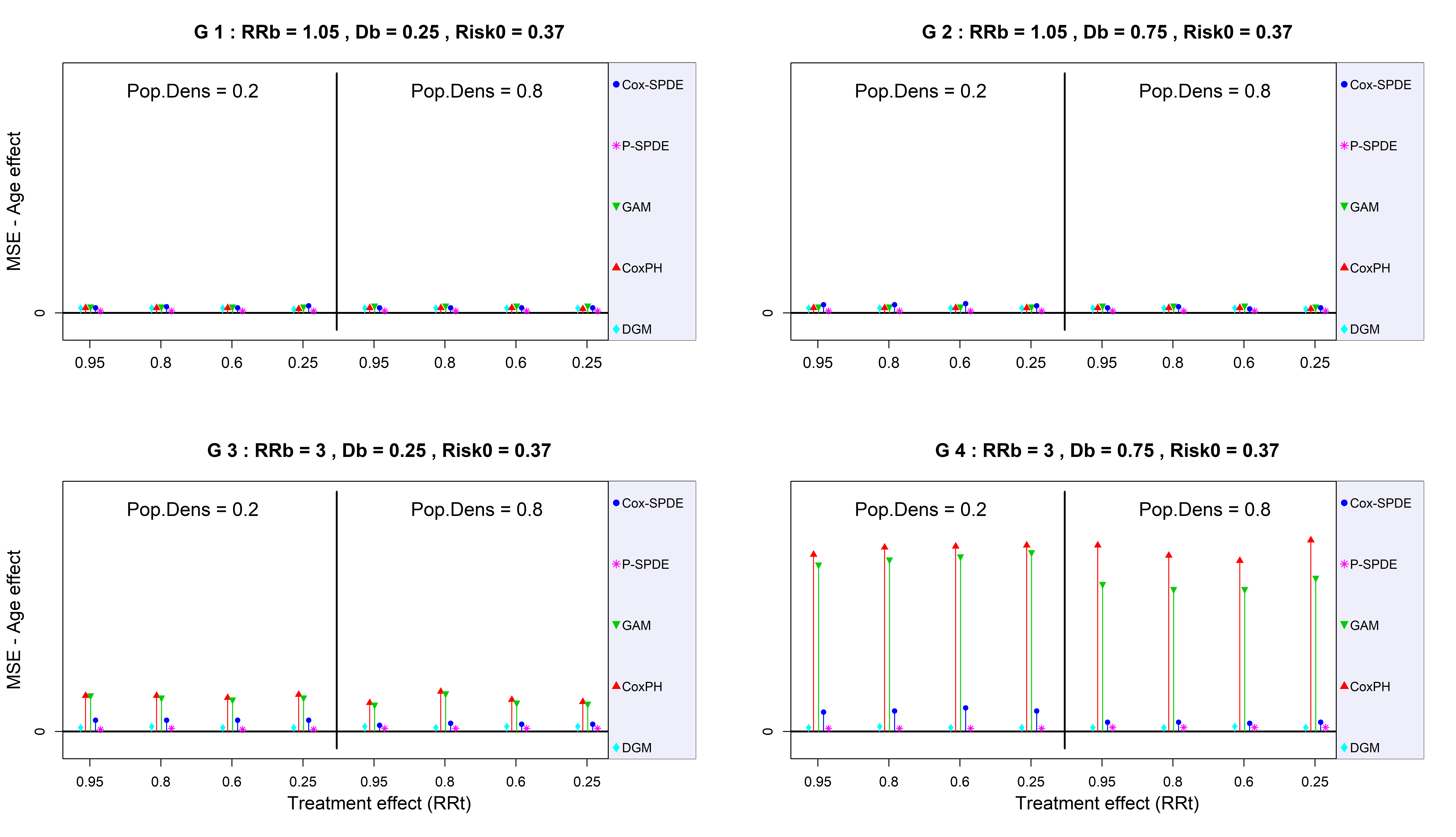

Supplement: Supplementary file 8 — Figure S5. MSE of the age effect with a baseline risk of 0.37.DGM: Data-Generating Model, Cox-PH: Cox Proportional Hazard model, GAM: Generalized Additive Model, Cox-SPDE: Cox-Stochastic Partial Differential Equation Model, P-SPDE: Poisson-Stochastic Partial Differential Equation, RRb: Breeding site Relative Risk, Db: Breeding site Density, RRt: Treatment Relative Risk, Pop.Dens: Population Density, Risk0: Baseline Risk. (TIF 753 kb) [file 12874_2019_759_MOESM8_ESM.tif]

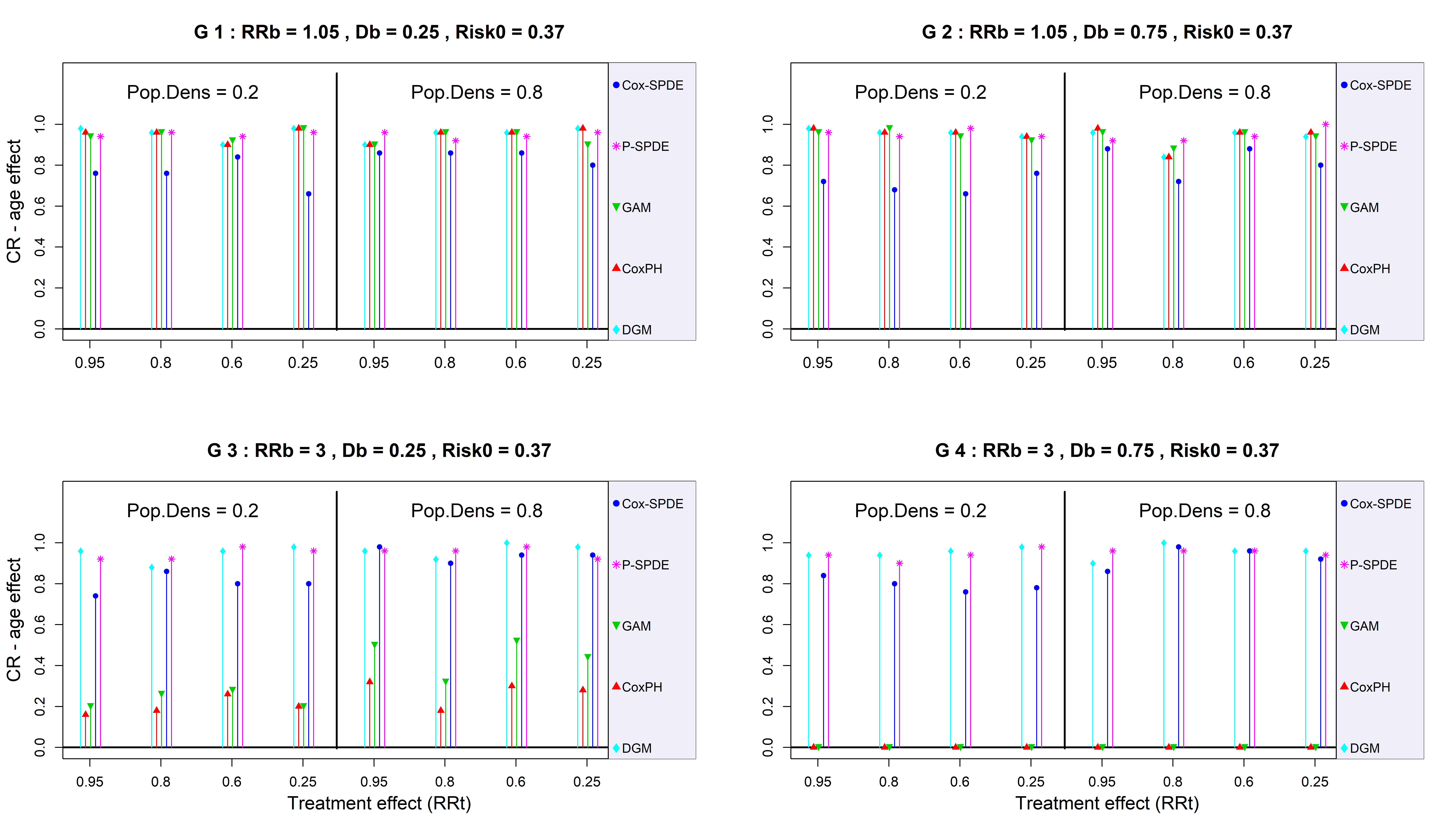

Supplement: Supplementary file 9 — Figure S6. CR of the age effect with a baseline risk of 0.37. DGM: Data-Generating Model, Cox-PH: Cox Proportional Hazard model, GAM: Generalized Additive Model, Cox-SPDE: Cox-Stochastic Partial Differential Equation Model, P-SPDE: Poisson-Stochastic Partial Differential Equation, RRb: Breeding site Relative Risk, Db: Breeding site Density, RRt: Treatment Relative Risk, Pop.Dens: Population Density, Risk0: Baseline Risk. (TIF 1247 kb) [file 12874_2019_759_MOESM9_ESM.tif]

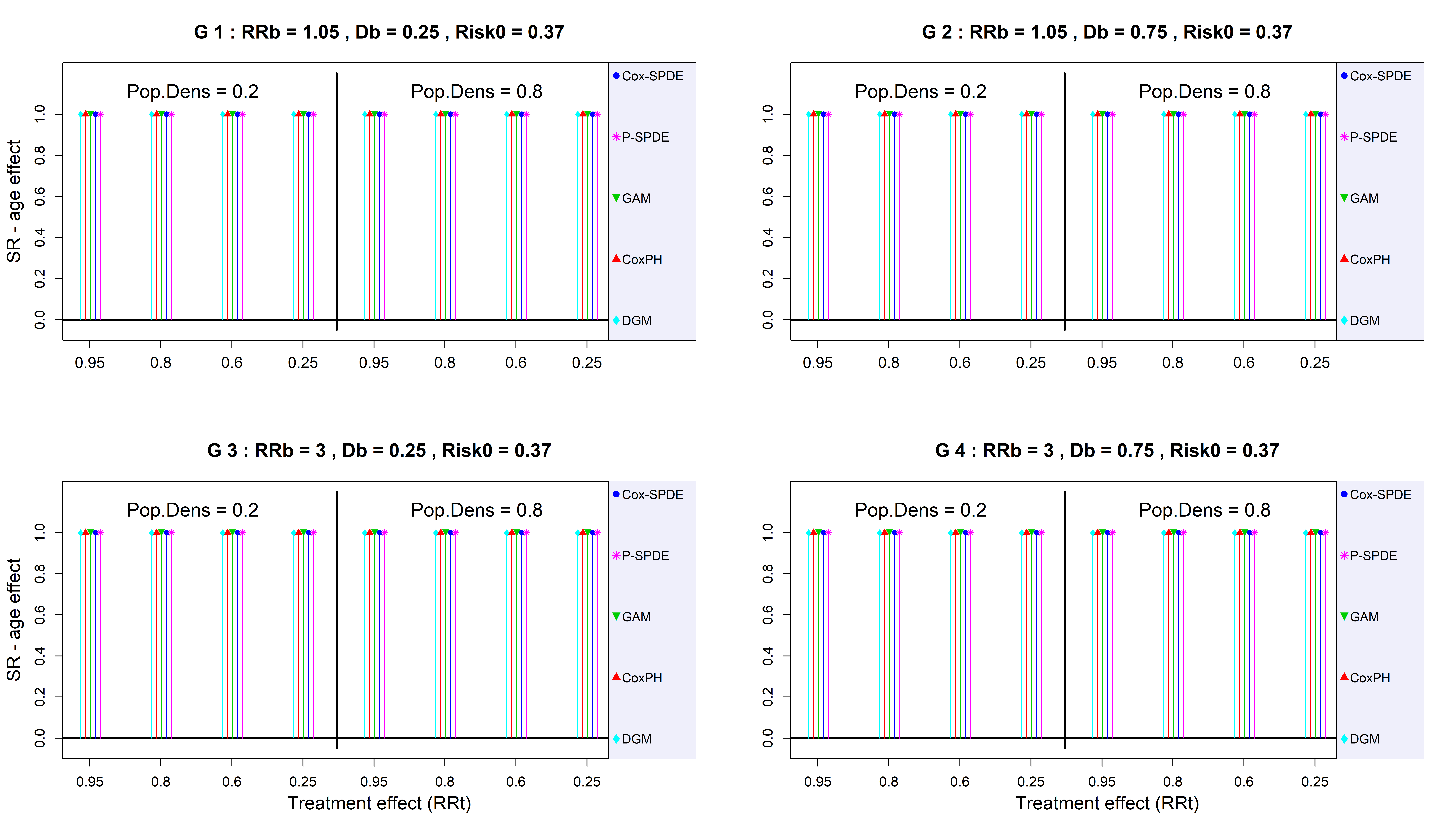

Supplement: Supplementary file 10 — Figure S7. SR of the age effect with a baseline risk of 0.37. DGM: Data-Generating Model, Cox-PH: Cox Proportional Hazard model, GAM: Generalized Additive Model, Cox-SPDE: Cox-Stochastic Partial Differential Equation Model, P-SPDE: Poisson-Stochastic Partial Differential Equation, RRb: Breeding site Relative Risk, Db: Breeding site Density, RRt: Treatment Relative Risk, Pop.Dens: Population Density, Risk0: Baseline Risk. (TIF 1407 kb) [file 12874_2019_759_MOESM10_ESM.tif]

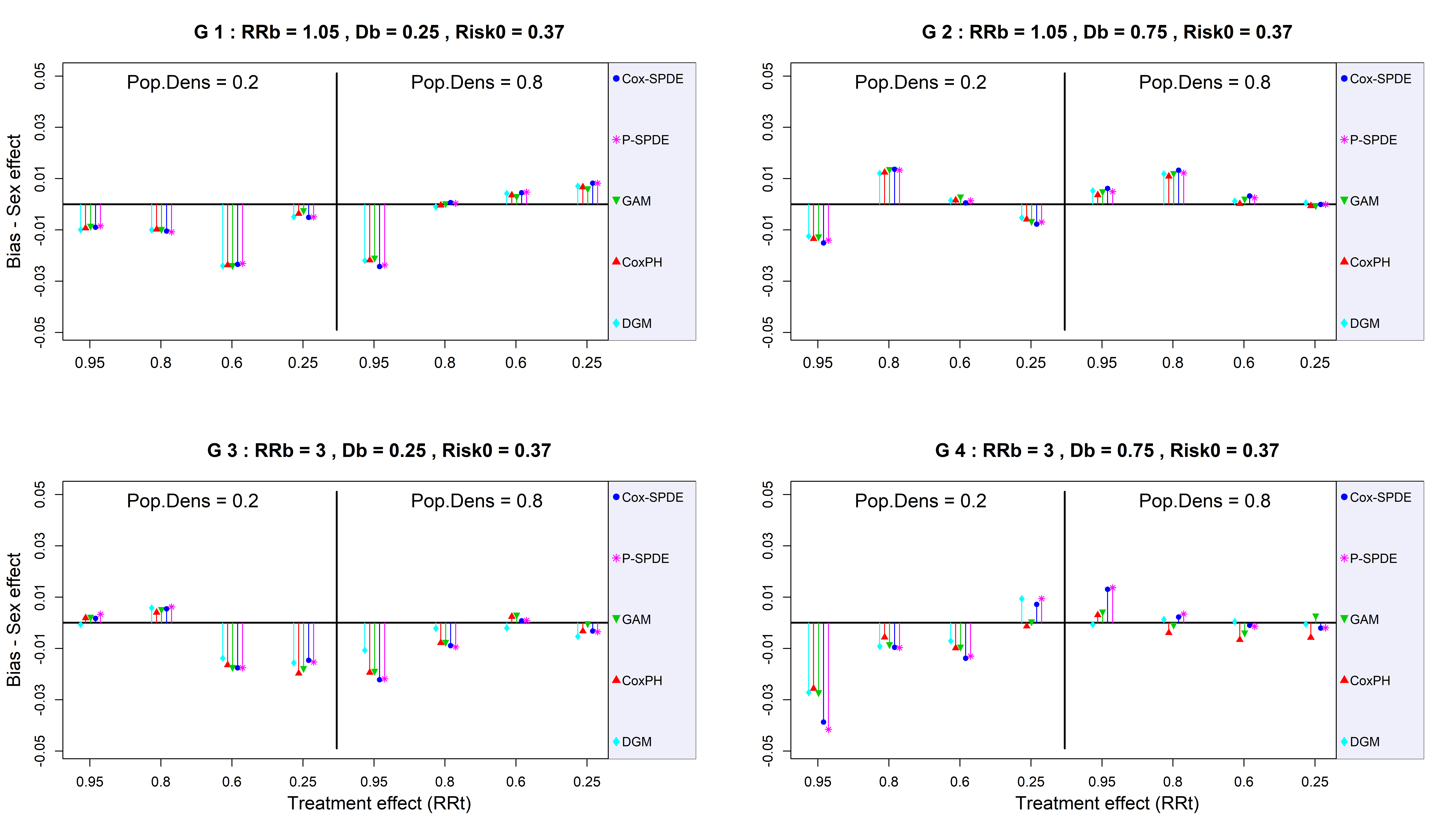

Supplement: Supplementary file 11 — Figure S8. Bias of the sex effect with a baseline risk of 0.37. DGM: Data-Generating Model, Cox-PH: Cox Proportional Hazard model, GAM: Generalized Additive Model, Cox-SPDE: Cox-Stochastic Partial Differential Equation Model, P-SPDE: Poisson-Stochastic Partial Differential Equation, RRb: Breeding site Relative Risk, Db: Breeding site Density, RRt: Treatment Relative Risk, Pop.Dens: Population Density, Risk0: Baseline Risk. (TIF 820 kb) [file 12874_2019_759_MOESM11_ESM.tif]

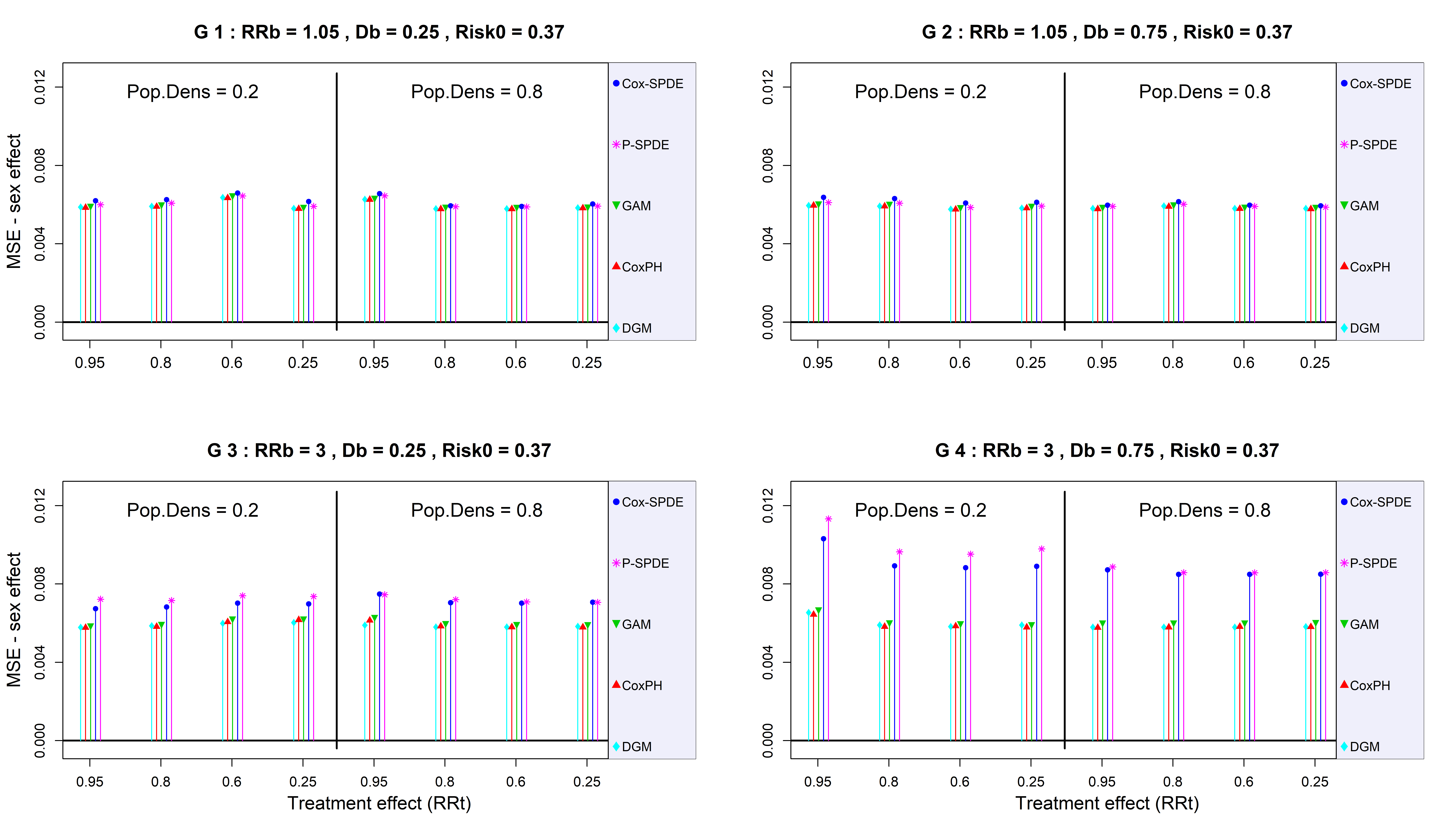

Supplement: Supplementary file 12 — Figure S9. MSE of the sex effect with a baseline risk of 0.37. DGM: Data-Generating Model, Cox-PH: Cox Proportional Hazard model, GAM: Generalized Additive Model, Cox-SPDE: Cox-Stochastic Partial Differential Equation Model, P-SPDE: Poisson-Stochastic Partial Differential Equation, RRb: Breeding site Relative Risk, Db: Breeding site Density, RRt: Treatment Relative Risk, Pop.Dens: Population Density, Risk0: Baseline Risk. (TIF 1147 kb) [file 12874_2019_759_MOESM12_ESM.tif]

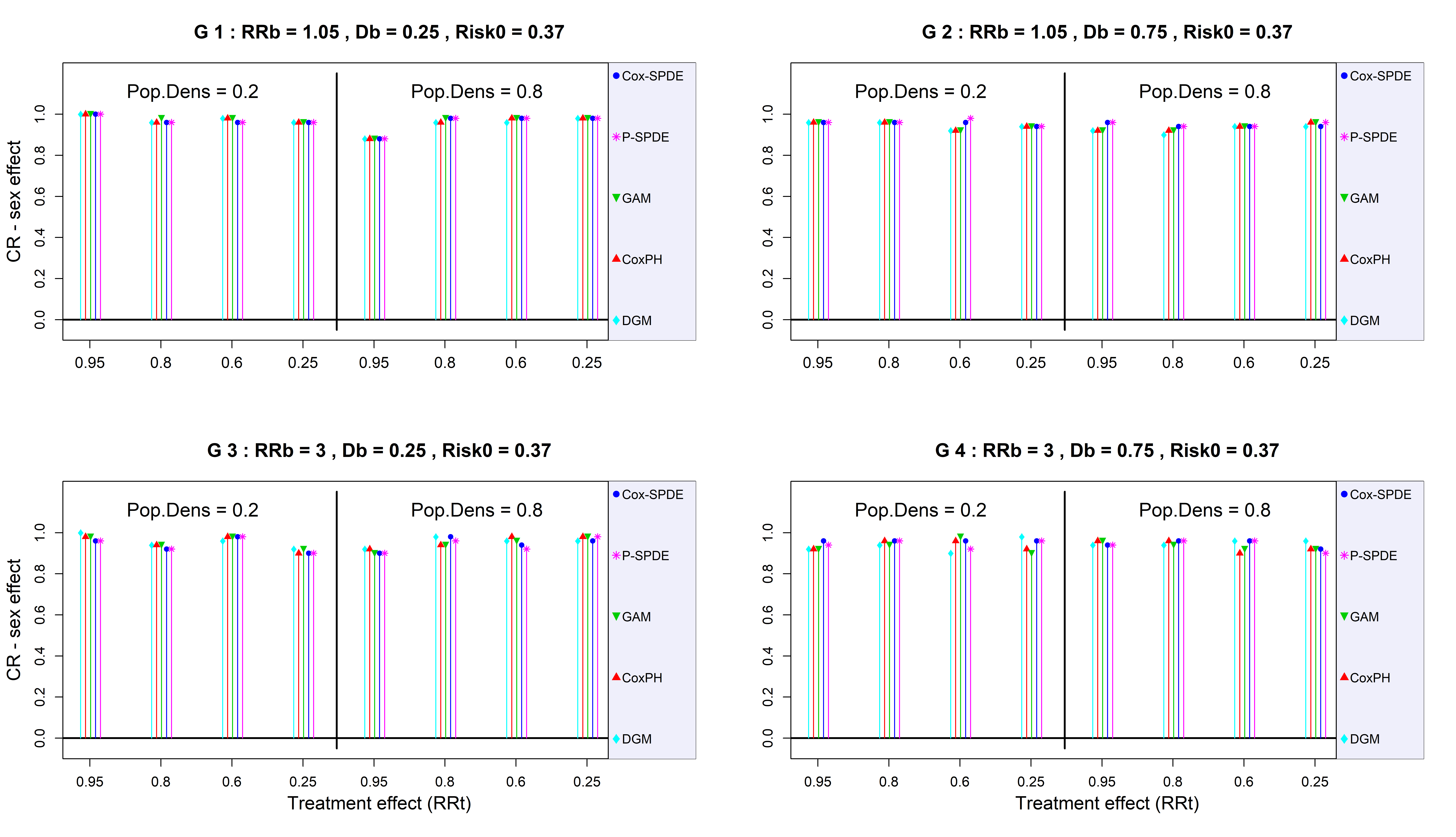

Supplement: Supplementary file 13 — Figure S10. CR of the sex effect with a baseline risk of 0.37. DGM: Data-Generating Model, Cox-PH: Cox Proportional Hazard model, GAM: Generalized Additive Model, Cox-SPDE: Cox-Stochastic Partial Differential Equation Model, P-SPDE: Poisson-Stochastic Partial Differential Equation, RRb: Breeding site Relative Risk, Db: Breeding site Density, RRt: Treatment Relative Risk, Pop.Dens: Population Density, Risk0: Baseline Risk. (TIF 1380 kb) [file 12874_2019_759_MOESM13_ESM.tif]

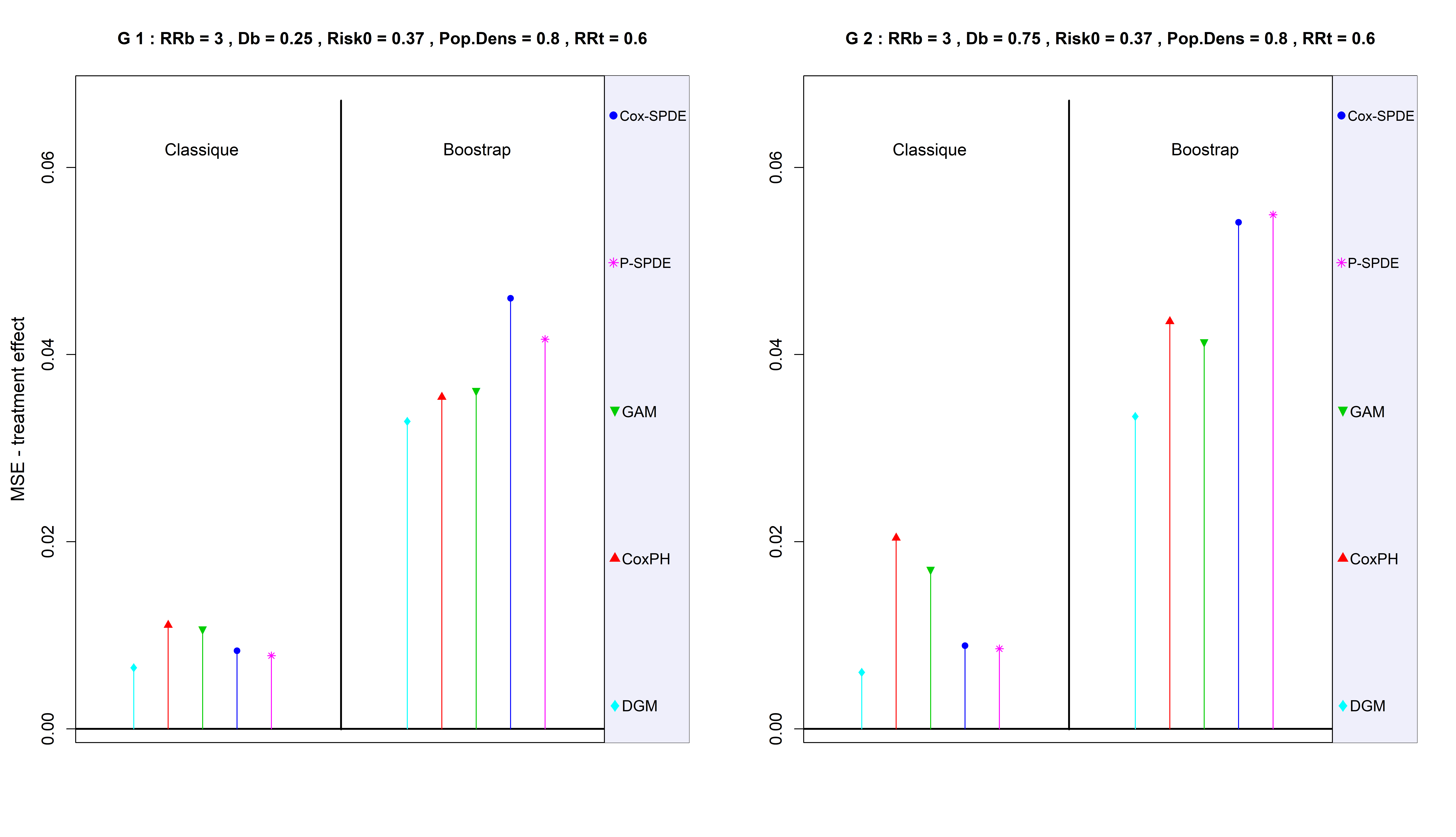

Supplement: Supplementary file 14 — Figure S11. MSE of the treatment effect with the classic and bootstrap methods. DGM: Data-Generating Model, Cox-PH: Cox Proportional Hazard model, GAM: Generalized Additive Model, Cox-SPDE: Cox-Stochastic Partial Differential Equation Model, P-SPDE: Poisson-Stochastic Partial Differential Equation, RRb: Breeding site Relative Risk, Db: Breeding site Density, RRt: Treatment Relative Risk, Pop.Dens: Population Density, Risk0: Baseline Risk. (TIF 650 kb) [file 12874_2019_759_MOESM14_ESM.tif]
