# Supplementary material for: “Spatial heterogeneity of environmental risk in randomized prevention trials: consequences and modeling”
Source: BMC Med Res Methodol. 2019 Jul 15;19:149. doi: 10.1186/s12874-019-0759-z (PMC6632226; doi:10.1186/s12874-019-0759-z)
Supplement: Supplementary file 2 — Description of mathematical formulas and terms used in this manuscript. (DOCX 17 kb) [file 12874_2019_759_MOESM2_ESM.docx]

**Cox Proportional Hazard model (CoxPH)**

The CoxPH model was originally designed to model survival. However, this model does not account for spatial heterogeneity, it is generally written as follows:

$$\lambda\left( t,X \right)=\lambda_{0}\left( t \right)\exp\left( \beta_{1}X_{1}+\beta_{2}X_{2}+\ldots+\beta_{n}X_{n} \right)$$

$\lambda\left( t,X \right)$ was the instant risk function at time $t,$ $\lambda_{0}\left( t \right)$was the baseline risk, constant over time, $X=\left( X_{1}, X_{2},\ldots, X_{n} \right)$was the fixed effect vector of covariates and $\beta=(\beta_{1}, \beta_{2},\ldots,\beta_{n})$was the vector of parameters related to each covariate.

This fixed-effect model was used to analyze the time of occurrence of an event of interest with the possibility of adjusting for covariates. Parameters were estimated with the maximum likelihood method.

**Model performance measures**

The parameter $\beta$ represented the true effect of each covariate estimated by $\hat{\beta}$, with a standard deviation $\bar{Sd\left( \hat{\beta} \right)}$estimated on $K$ datasets for each scenario and with a 95% Confidence Interval $IC\left( \beta\right).$

$$\bar{Sd\left( \hat{\beta} \right)}=\frac{1}{K}\sum_{i=1}^{K} Sd\left( \hat{\beta}_{i} \right)$$

**Bias**

$B\left( \hat{\beta} \right)$was an indicator of estimator performance expressing the difference between true effect and estimated effect:

$$B\left( \hat{\beta} \right)=\bar{\hat{\beta}}-\beta$$

$\bar{\hat{\beta}}=1/K\sum_{i=1}^{K} \hat{\beta}_{i}$was the estimated average effect for a scenario based on the 50 datasets.

**Mean Square Error (MSE)**

The MSE was used to measure the stability and accuracy of the estimates:

$$MSE\left( \hat{\beta} \right)=\left( B\left( \hat{\beta} \right) \right)^{2}+ \left( \bar{Sd\left( \hat{\beta} \right)} \right)^{2}$$

**Coverage Rate (CR)**

The CR estimated the number of times the true effect was covered by the 95% Confidence Interval:

$$CR(\beta)=\frac{1}{K}\sum_{i=1}^{K} \mathbb{1}_{\left\{ \beta\epsilon IC\left( \hat{\beta} \right) \right\}}$$

**Significance rate (SR)**

The SR expressed the proportion of the number of times the estimated effect was significant, *i.e.*, the number of times the Confidence Interval of the parameters did not contain the zero value:

$$SR(\beta)=\frac{1}{K}\sum_{i=1}^{K} \mathbb{1}_{\left\{ 0 \notin IC\left( \hat{\beta} \right) \right\}}$$

**Matèrn covariance function**

The Matèrn covariance function was used to control for spatial dependence between two individuals $i$ and $j$ as a function of the Euclidean distance $d_{i,j}$ between them:

$\Sigma_{ij}\left( \theta_{1},\theta_{2} \right)$= $\sigma_{X}Cov\left( X_{i},X_{j} \right)=\sigma_{X}\frac{2^{1-\theta_{1}}}{\Gamma\left( \theta_{1} \right)}\left( \theta_{2}d_{i,j} \right)^{\theta_{1}}K_{\theta_{1}}\left( \theta_{2}d_{i,j} \right)$

$\sigma_{X}$was the marginal standard deviation of the variable $X$ being studied, $\Gamma$ was a gamma function, $K_{\theta_{1}}$ was a Bessel function, $\theta_{1} \mathrm{and} \theta_{2}$ were the spatial decay parameters of the Matèrn function.
