# Supplementary material for: “Spatial heterogeneity of environmental risk in randomized prevention trials: consequences and modeling”
Source: BMC Med Res Methodol. 2019 Jul 15;19:149. doi: 10.1186/s12874-019-0759-z (PMC6632226; doi:10.1186/s12874-019-0759-z)
Supplement: Supplementary file 3 — Data-Generating Model (DGM) performance index for estimating the different factor effects (baseline risk 0.37). (DOCX 23 kb) [file 12874_2019_759_MOESM3_ESM.docx]

Table A.2: Data-Generating Model (DGM) performance index for estimating the different factor effects (baseline risk 0.37).

| RRb | RRt | factors | Breeding site density | | | | | | | | | | | | | | | | | |
| --- | --- | --- | --- | --- | --- | --- | --- | --- | --- | --- | --- | --- | --- | --- | --- | --- | --- | --- | --- | --- |
|  |  |  | 0.25 | | | | | | | | | 0.75 | | | | | | | | |
|  |  |  | Population density | | | | | | | | | Population density | | | | | | | | |
|  |  |  | 0.2 | | | | 0.8 | | | | | 0.2 | | | | | 0.8 | | | |
|  |  |  | Bias | MSE | CR | SR | Bias | MSE | CR | SR | Bias | | MSE | CR | SR | Bias | | MSE | CR | SR |
| 1.05 | 0.95 | Age | 0.001 | 0.000 | 0.98 | 1.00 | 0.000 | 0.000 | 0.90 | 1.00 | -0.002 | | 0.000 | 0.98 | 1.00 | -0.001 | | 0.000 | 0.96 | 1.00 |
|  |  | Sex | -0.010 | 0.006 | 1.00 | 0.00 | -0.022 | 0.006 | 0.88 | 0.12 | -0.012 | | 0.006 | 0.96 | 0.04 | 0.005 | | 0.006 | 0.92 | 0.08 |
|  |  | Treatment | 0.015 | 0.006 | 0.96 | 0.06 | 0.014 | 0.006 | 0.84 | 0.06 | 0.015 | | 0.006 | 0.92 | 0.06 | 0.000 | | 0.006 | 0.96 | 0.04 |
|  |  | BS | 0.011 | 0.006 | 0.94 | 0.12 | -0.011 | 0.006 | 0.92 | 0.14 | 0.006 | | 0.002 | 1.00 | 0.26 | 0.007 | | 0.002 | 0.94 | 0.26 |
|  | 0.80 | Age | 0.000 | 0.000 | 0.96 | 1.00 | -0.002 | 0.000 | 0.96 | 1.00 | -0.001 | | 0.000 | 0.96 | 1.00 | -0.003 | | 0.000 | 0.84 | 1.00 |
|  |  | Sex | -0.010 | 0.006 | 0.96 | 0.04 | -0.001 | 0.006 | 0.96 | 0.04 | 0.012 | | 0.006 | 0.96 | 0.04 | 0.012 | | 0.006 | 0.9 | 0.10 |
|  |  | Treatment | -0.012 | 0.006 | 0.94 | 0.74 | -0.005 | 0.006 | 0.98 | 0.92 | -0.018 | | 0.006 | 0.96 | 0.92 | 0.013 | | 0.006 | 0.96 | 0.76 |
|  |  | BS | 0.007 | 0.006 | 0.92 | 0.10 | 0.019 | 0.007 | 0.92 | 0.10 | -0.010 | | 0.002 | 0.98 | 0.10 | -0.004 | | 0.002 | 0.96 | 0.16 |
|  | 0.60 | Age | 0.001 | 0.000 | 0.90 | 1.00 | -0.001 | 0.000 | 0.96 | 1.00 | -0.002 | | 0.000 | 0.96 | 1.00 | 0.000 | | 0.000 | 0.96 | 1.00 |
|  |  | Sex | -0.024 | 0.006 | 0.98 | 0.02 | 0.004 | 0.006 | 0.96 | 0.04 | 0.002 | | 0.006 | 0.92 | 0.08 | 0.001 | | 0.006 | 0.94 | 0.06 |
|  |  | Treatment | -0.004 | 0.006 | 0.88 | 1.00 | -0.006 | 0.006 | 0.96 | 1.00 | -0.016 | | 0.006 | 0.94 | 1.00 | -0.008 | | 0.006 | 0.94 | 1.00 |
|  |  | BS | 0.008 | 0.006 | 0.98 | 0.14 | -0.005 | 0.007 | 0.96 | 0.12 | -0.004 | | 0.002 | 0.96 | 0.16 | -0.002 | | 0.002 | 0.94 | 0.18 |
|  | 0.25 | Age | 0.000 | 0.000 | 0.98 | 1.00 | -0.001 | 0.000 | 0.98 | 1.00 | -0.001 | | 0.000 | 0.94 | 1.00 | 0.000 | | 0.000 | 0.94 | 1.00 |
|  |  | Sex | -0.005 | 0.006 | 0.96 | 0.04 | 0.007 | 0.006 | 0.98 | 0.02 | -0.005 | | 0.006 | 0.94 | 0.06 | 0.001 | | 0.006 | 0.94 | 0.06 |
|  |  | Treatment | 0.007 | 0.007 | 0.98 | 1.00 | -0.014 | 0.008 | 0.98 | 1.00 | -0.020 | | 0.008 | 0.96 | 1.00 | 0.011 | | 0.008 | 0.9 | 1.00 |
|  |  | BS | 0.013 | 0.006 | 0.98 | 0.08 | -0.008 | 0.007 | 0.90 | 0.12 | -0.002 | | 0.002 | 0.94 | 0.18 | -0.005 | | 0.002 | 0.92 | 0.24 |
| 3 | 0.95 | Age | 0.001 | 0.000 | 0.96 | 1.00 | -0.001 | 0.000 | 0.96 | 1.00 | -0.001 | | 0.000 | 0.94 | 1.00 | 0.000 | | 0.000 | 0.9 | 1.00 |
|  |  | Sex | -0.001 | 0.006 | 1.00 | 0.00 | -0.011 | 0.006 | 0.92 | 0.08 | -0.027 | | 0.007 | 0.92 | 0.08 | -0.001 | | 0.006 | 0.94 | 0.06 |
|  |  | Treatment | 0.001 | 0.006 | 1.00 | 0.04 | -0.006 | 0.006 | 0.94 | 0.10 | 0.018 | | 0.006 | 0.96 | 0.10 | 0.004 | | 0.006 | 0.94 | 0.06 |
|  |  | BS | 0.014 | 0.007 | 0.88 | 1.00 | -0.012 | 0.007 | 0.94 | 1.00 | 0.001 | | 0.003 | 0.94 | 1.00 | 0.005 | | 0.003 | 0.92 | 1.00 |
|  | 0.80 | Age | -0.001 | 0.000 | 0.88 | 1.00 | 0.001 | 0.000 | 0.92 | 1.00 | -0.002 | | 0.000 | 0.94 | 1.00 | -0.001 | | 0.000 | 1.00 | 1.00 |
|  |  | Sex | 0.006 | 0.006 | 0.94 | 0.06 | -0.002 | 0.006 | 0.98 | 0.02 | -0.009 | | 0.006 | 0.94 | 0.06 | 0.001 | | 0.006 | 0.94 | 0.06 |
|  |  | Treatment | -0.018 | 0.006 | 0.96 | 0.88 | -0.007 | 0.006 | 0.98 | 0.86 | -0.008 | | 0.006 | 0.94 | 0.88 | 0.008 | | 0.006 | 0.96 | 0.72 |
|  |  | BS | 0.017 | 0.007 | 0.98 | 1.00 | 0.021 | 0.007 | 0.96 | 1.00 | 0.016 | | 0.003 | 0.90 | 1.00 | 0.008 | | 0.003 | 0.96 | 1.00 |
|  | 0.60 | Age | 0.000 | 0.000 | 0.96 | 1.00 | -0.001 | 0.000 | 1.00 | 1.00 | 0.000 | | 0.000 | 0.96 | 1.00 | -0.002 | | 0.000 | 0.96 | 1.00 |
|  |  | Sex | -0.014 | 0.006 | 0.96 | 0.04 | -0.002 | 0.006 | 0.96 | 0.04 | -0.007 | | 0.006 | 0.90 | 0.10 | 0.001 | | 0.006 | 0.96 | 0.04 |
|  |  | Treatment | -0.011 | 0.006 | 0.98 | 1.00 | 0.023 | 0.007 | 0.92 | 1.00 | 0.004 | | 0.006 | 0.90 | 1.00 | -0.006 | | 0.006 | 0.94 | 1.00 |
|  |  | BS | 0.004 | 0.007 | 0.96 | 1.00 | 0.012 | 0.007 | 1.00 | 1.00 | 0.006 | | 0.003 | 0.98 | 1.00 | 0.007 | | 0.003 | 0.96 | 1.00 |
|  | 0.25 | Age | 0.000 | 0.000 | 0.98 | 1.00 | -0.002 | 0.000 | 0.98 | 1.00 | 0.000 | | 0.000 | 0.98 | 1.00 | -0.001 | | 0.000 | 0.96 | 1.00 |
|  |  | Sex | -0.016 | 0.006 | 0.92 | 0.08 | -0.005 | 0.006 | 0.96 | 0.04 | 0.009 | | 0.006 | 0.98 | 0.02 | 0.000 | | 0.006 | 0.96 | 0.04 |
|  |  | Treatment | -0.012 | 0.008 | 1.00 | 1.00 | -0.007 | 0.007 | 0.98 | 1.00 | -0.001 | | 0.007 | 0.96 | 1.00 | -0.007 | | 0.007 | 0.98 | 1.00 |
|  |  | BS | 0.003 | 0.007 | 0.96 | 1.00 | 0.022 | 0.007 | 0.98 | 1.00 | 0.005 | | 0.003 | 0.98 | 1.00 | 0.008 | | 0.003 | 0.96 | 1.00 |

**BS**: Breeding site**, CR**: Coverage Rate, **SR**: Significance Rate**, RRt**: Treatment Relative Risk, **RRb**: Breeding site Relative Risk.
