# Supplementary material for: “Spatial heterogeneity of environmental risk in randomized prevention trials: consequences and modeling”
Source: BMC Med Res Methodol. 2019 Jul 15;19:149. doi: 10.1186/s12874-019-0759-z (PMC6632226; doi:10.1186/s12874-019-0759-z)
Supplement: Supplementary file 6 — MSE of the treatment, age, and sex effect for all models (baseline risk 0.37). (DOCX 32 kb) [file 12874_2019_759_MOESM6_ESM.docx]

Table 4: MSE of the treatment effect for all models (baseline risk 0.37)

| RRb | RRt | Model | Breeding site density | | | |
| --- | --- | --- | --- | --- | --- | --- |
|  |  |  | 0.25 | | 0.75 | |
|  |  |  | Population density | | Population density | |
|  |  |  | 0.2 | 0.8 | 0.2 | 0.8 |
| 1.05 | 0.95 | CoxPH | 0.006 | 0.006 | 0.006 | 0.0058 |
|  |  | GAM | 0.006 | 0.006 | 0.006 | 0.0058 |
|  |  | Cox-SPDE | 0.0063 | 0.0062 | 0.0063 | 0.0059 |
|  |  | P-SPDE | 0.0061 | 0.0061 | 0.0061 | 0.0059 |
|  | 0.80 | CoxPH | 0.006 | 0.0058 | 0.0061 | 0.006 |
|  |  | GAM | 0.006 | 0.0059 | 0.0062 | 0.006 |
|  |  | Cox-SPDE | 0.0065 | 0.006 | 0.0069 | 0.0061 |
|  |  | P-SPDE | 0.0061 | 0.0059 | 0.0062 | 0.0061 |
|  | 0.60 | CoxPH | 0.006 | 0.006 | 0.0062 | 0.0061 |
|  |  | GAM | 0.006 | 0.0061 | 0.0063 | 0.0061 |
|  |  | Cox-SPDE | 0.0065 | 0.0062 | 0.007 | 0.0064 |
|  |  | P-SPDE | 0.006 | 0.0059 | 0.006 | 0.006 |
|  | 0.25 | CoxPH | 0.0075 | 0.0076 | 0.0078 | 0.0076 |
|  |  | GAM | 0.0075 | 0.0078 | 0.0079 | 0.0075 |
|  |  | Cox-SPDE | 0.0091 | 0.0085 | 0.0107 | 0.0074 |
|  |  | P-SPDE | 0.0061 | 0.0061 | 0.0061 | 0.0063 |
| 3 | 0.95 | CoxPH | 0.0058 | 0.0058 | 0.0065 | 0.0058 |
|  |  | GAM | 0.0058 | 0.0059 | 0.0066 | 0.006 |
|  |  | Cox-SPDE | 0.0068 | 0.007 | 0.0094 | 0.0085 |
|  |  | P-SPDE | 0.0072 | 0.007 | 0.0101 | 0.0087 |
|  | 0.80 | CoxPH | 0.006 | 0.0061 | 0.0079 | 0.0106 |
|  |  | GAM | 0.006 | 0.0061 | 0.0078 | 0.0106 |
|  |  | Cox-SPDE | 0.0068 | 0.007 | 0.009 | 0.0091 |
|  |  | P-SPDE | 0.0072 | 0.0072 | 0.0096 | 0.0091 |
|  | 0.60 | CoxPH | 0.0073 | 0.0111 | 0.0252 | 0.0204 |
|  |  | GAM | 0.0072 | 0.0105 | 0.0241 | 0.0169 |
|  |  | Cox-SPDE | 0.0071 | 0.0084 | 0.012 | 0.0089 |
|  |  | P-SPDE | 0.0072 | 0.0078 | 0.0101 | 0.0086 |
|  | 0.25 | CoxPH | 0.0228 | 0.0256 | 0.1091 | 0.1155 |
|  |  | GAM | 0.0218 | 0.0222 | 0.1025 | 0.0928 |
|  |  | Cox-SPDE | 0.0116 | 0.0098 | 0.0177 | 0.0118 |
|  |  | P-SPDE | 0.0074 | 0.0072 | 0.0104 | 0.0088 |

**MSE**: Mean Square Error, **CR**: Coverage Rate, **SR**: Significance Rate, **CoxPH**: Cox Proportional Hazard model, **GAM**: Generalized Additive Model, **Cox-SPDE**: Cox Stochastic Partial Differential Equation Model, **P-SPDE**: Poisson Stochastic Partial Differential Equation Model**, RRt**: Treatment Relative Risk, **RRb**: Breeding site Relative Risk

Table 5: MSE of the age effect for all models (baseline risk 0.37)

| RRb | RRt | Model | Breeding site density | | | |
| --- | --- | --- | --- | --- | --- | --- |
|  |  |  | 0.25 | | 0.75 | |
|  |  |  | Population density | | Population density | |
|  |  |  | 0.2 | 0.8 | 0.2 | 0.8 |
| 1.05 | 0.95 | CoxPH | < 0.0001 | < 0.0001 | < 0.0001 | < 0.0001 |
|  |  | GAM | < 0.0001 | 0.0001 | < 0.0001 | 0.0001 |
|  |  | Cox-SPDE | < 0.0001 | < 0.0001 | 0.0001 | < 0.0001 |
|  |  | P-SPDE | < 0.0001 | < 0.0001 | < 0.0001 | < 0.0001 |
|  | 0.80 | CoxPH | < 0.0001 | < 0.0001 | < 0.0001 | < 0.0001 |
|  |  | GAM | < 0.0001 | 0.0001 | < 0.0001 | 0.0001 |
|  |  | Cox-SPDE | 0.0001 | < 0.0001 | 0.0001 | 0.0001 |
|  |  | P-SPDE | < 0.0001 | < 0.0001 | < 0.0001 | < 0.0001 |
|  | 0.60 | CoxPH | < 0.0001 | < 0.0001 | < 0.0001 | < 0.0001 |
|  |  | GAM | < 0.0001 | 0.0001 | < 0.0001 | 0.0001 |
|  |  | Cox-SPDE | < 0.0001 | < 0.0001 | 0.0001 | < 0.0001 |
|  |  | P-SPDE | < 0.0001 | < 0.0001 | < 0.0001 | < 0.0001 |
|  | 0.25 | CoxPH | < 0.0001 | < 0.0001 | < 0.0001 | < 0.0001 |
|  |  | GAM | < 0.0001 | 0.0001 | < 0.0001 | < 0.0001 |
|  |  | Cox-SPDE | 0.0001 | < 0.0001 | 0.0001 | < 0.0001 |
|  |  | P-SPDE | < 0.0001 | < 0.0001 | < 0.0001 | < 0.0001 |
| 3 | 0.95 | CoxPH | 0.0004 | 0.0003 | 0.0017 | 0.0018 |
|  |  | GAM | 0.0003 | 0.0002 | 0.0016 | 0.0014 |
|  |  | Cox-SPDE | 0.0001 | 0.0001 | 0.0002 | 0.0001 |
|  |  | P-SPDE | < 0.0001 | < 0.0001 | < 0.0001 | < 0.0001 |
|  | 0.80 | CoxPH | 0.0004 | 0.0004 | 0.0018 | 0.0017 |
|  |  | GAM | 0.0003 | 0.0004 | 0.0017 | 0.0014 |
|  |  | Cox-SPDE | 0.0001 | 0.0001 | 0.0002 | 0.0001 |
|  |  | P-SPDE | < 0.0001 | < 0.0001 | < 0.0001 | < 0.0001 |
|  | 0.60 | CoxPH | 0.0003 | 0.0003 | 0.0018 | 0.0017 |
|  |  | GAM | 0.0003 | 0.0003 | 0.0017 | 0.0014 |
|  |  | Cox-SPDE | 0.0001 | 0.0001 | 0.0002 | 0.0001 |
|  |  | P-SPDE | < 0.0001 | < 0.0001 | < 0.0001 | < 0.0001 |
|  | 0.25 | CoxPH | 0.0004 | 0.0003 | 0.0018 | 0.0019 |
|  |  | GAM | 0.0003 | 0.0003 | 0.0017 | 0.0015 |
|  |  | Cox-SPDE | 0.0001 | 0.0001 | 0.0002 | 0.0001 |
|  |  | P-SPDE | < 0.0001 | < 0.0001 | < 0.0001 | < 0.0001 |

**MSE**: Mean Square Error, **CR**: Coverage Rate, **SR**: Significance Rate, **CoxPH**: Cox Proportional Hazard model, **GAM**: Generalized Additive Model, **Cox-SPDE**: Cox Stochastic Partial Differential Equation Model, **P-SPDE**: Poisson Stochastic Partial Differential Equation Model**, RRt**: Treatment Relative Risk, **RRb**: Breeding site Relative Risk

Table 6: MSE of the sex effect for all models (baseline risk 0.37)

| RRb | RRt | Model | Breeding site density | | | |
| --- | --- | --- | --- | --- | --- | --- |
|  |  |  | 0.25 | | 0.75 | |
|  |  |  | Population density | | Population density | |
|  |  |  | 0.2 | 0.8 | 0.2 | 0.8 |
| 1.05 | 0.95 | CoxPH | 0.0058 | 0.0063 | 0.006 | 0.0058 |
|  |  | GAM | 0.0059 | 0.0063 | 0.006 | 0.0058 |
|  |  | Cox-SPDE | 0.0062 | 0.0066 | 0.0064 | 0.006 |
|  |  | P-SPDE | 0.006 | 0.0064 | 0.0061 | 0.0059 |
|  | 0.80 | CoxPH | 0.0059 | 0.0058 | 0.0059 | 0.0059 |
|  |  | GAM | 0.006 | 0.0058 | 0.006 | 0.006 |
|  |  | Cox-SPDE | 0.0062 | 0.0059 | 0.0063 | 0.0062 |
|  |  | P-SPDE | 0.0061 | 0.0059 | 0.0061 | 0.006 |
|  | 0.60 | CoxPH | 0.0063 | 0.0058 | 0.0058 | 0.0058 |
|  |  | GAM | 0.0064 | 0.0058 | 0.0058 | 0.0058 |
|  |  | Cox-SPDE | 0.0066 | 0.0059 | 0.0061 | 0.006 |
|  |  | P-SPDE | 0.0064 | 0.0059 | 0.0058 | 0.0059 |
|  | 0.25 | CoxPH | 0.0058 | 0.0058 | 0.0058 | 0.0058 |
|  |  | GAM | 0.0058 | 0.0058 | 0.0059 | 0.0058 |
|  |  | Cox-SPDE | 0.0062 | 0.006 | 0.0061 | 0.0059 |
|  |  | P-SPDE | 0.0059 | 0.0059 | 0.0059 | 0.0059 |
| 3 | 0.95 | CoxPH | 0.0058 | 0.0061 | 0.0064 | 0.0058 |
|  |  | GAM | 0.0058 | 0.0062 | 0.0066 | 0.006 |
|  |  | Cox-SPDE | 0.0067 | 0.0075 | 0.0103 | 0.0087 |
|  |  | P-SPDE | 0.0072 | 0.0074 | 0.0113 | 0.0089 |
|  | 0.80 | CoxPH | 0.0058 | 0.0058 | 0.0058 | 0.0058 |
|  |  | GAM | 0.0059 | 0.0059 | 0.006 | 0.006 |
|  |  | Cox-SPDE | 0.0068 | 0.007 | 0.0089 | 0.0085 |
|  |  | P-SPDE | 0.0072 | 0.0072 | 0.0096 | 0.0086 |
|  | 0.60 | CoxPH | 0.0061 | 0.0058 | 0.0058 | 0.0058 |
|  |  | GAM | 0.0062 | 0.0059 | 0.0059 | 0.006 |
|  |  | Cox-SPDE | 0.007 | 0.007 | 0.0088 | 0.0085 |
|  |  | P-SPDE | 0.0074 | 0.0071 | 0.0095 | 0.0086 |
|  | 0.25 | CoxPH | 0.0062 | 0.0058 | 0.0058 | 0.0058 |
|  |  | GAM | 0.0062 | 0.0059 | 0.0059 | 0.006 |
|  |  | Cox-SPDE | 0.007 | 0.0071 | 0.0089 | 0.0085 |
|  |  | P-SPDE | 0.0074 | 0.0071 | 0.0098 | 0.0086 |

**MSE**: Mean Square Error, **CR**: Coverage Rate, **SR**: Significance Rate, **CoxPH**: Cox Proportional Hazard model, **GAM**: Generalized Additive Model, **Cox-SPDE**: Cox Stochastic Partial Differential Equation Model, **P-SPDE**: Poisson Stochastic Partial Differential Equation Model**, RRt**: Treatment Relative Risk, **RRb**: Breeding site Relative Risk
